# Longitudinal studies of child mental disorders in the general population: A systematic review of study characteristics

**DOI:** 10.1002/jcv2.12186

**Published:** 2023-08-11

**Authors:** Theodora Bogdan, Weiyi Xie, Habeba Talaat, Hafsa Mir, Bhargavi Venkataraman, Laura E. Banfield, Katholiki Georgiades, Laura Duncan

**Affiliations:** ^1^ Department of Psychiatry and Behavioural Neurosciences Offord Centre for Child Studies McMaster University Hamilton Ontario Canada; ^2^ School of Nursing McMaster University Hamilton Ontario Canada

**Keywords:** children, epidemiologic studies, longitudinal studies, mental health, study design, systematic review

## Abstract

**Introduction:**

Longitudinal studies of child mental disorders in the general population (herein study) investigate trends in prevalence, incidence, risk/protective factors, and sequelae for disorders. They are time and resource intensive but offer life‐course perspectives and examination of causal mechanisms. Comprehensive syntheses of the methods of existing studies will provide an understanding of studies conducted to date, inventory studies, and inform the planning of new longitudinal studies.

**Methods:**

A systematic review of the research literature in MEDLINE, EMBASE, and PsycINFO was conducted in December 2022 for longitudinal studies of child mental disorders in the general population. Records were grouped by study and assessed for eligibility. Data were extracted from one of four sources: a record reporting study methodology, a record documenting child mental disorder prevalence, study websites, or user guides. Narrative and tabular syntheses of the scope and design features of studies were generated.

**Results:**

There were 18,133 unique records for 487 studies—159 of these were eligible for inclusion. Studies occurred from 1934 to 2019 worldwide, with data collection across 1 to 68 time points, with 70% of studies ongoing. Baseline sample sizes ranged from *n* = 151 to 64,136. Studies were most frequently conducted in the United States and at the city/town level. Internalizing disorders and disruptive, impulse control, and conduct disorders were the most frequently assessed mental disorders. Of studies reporting methods of disorder assessment, almost all used measurement scales. Individual, familial and environmental risk and protective factors and sequelae were examined.

**Conclusions:**

These results summarize characteristics of existing longitudinal studies of child mental disorders in the general population, provide an understanding of studies conducted to date, encourage comprehensive and consistent reporting of study methodology to facilitate meta‐analytic syntheses of longitudinal evidence, and offer recommendations and suggestions for the design of future studies. Registration DOI: 10.17605/OSF.IO/73HSW.

## Graphical abstract

Longitudinal studies of child mental disorders in the general population (herein study) investigate trends in prevalence, incidence, risk/protective factors, and sequelae for disorders. Studies occurred from 1934 to 2019 worldwide, with data collection across 1 to 68 time points, with 70% of studies ongoing. These results summarize characteristics of 159 longitudinal studies of child mental disorders in the general population, provide an understanding of studies conducted to date, encourage comprehensive and consistent reporting of study methodology to facilitate meta‐analytic syntheses of longitudinal evidence, and offer recommendations and suggestions for the design of future studies.

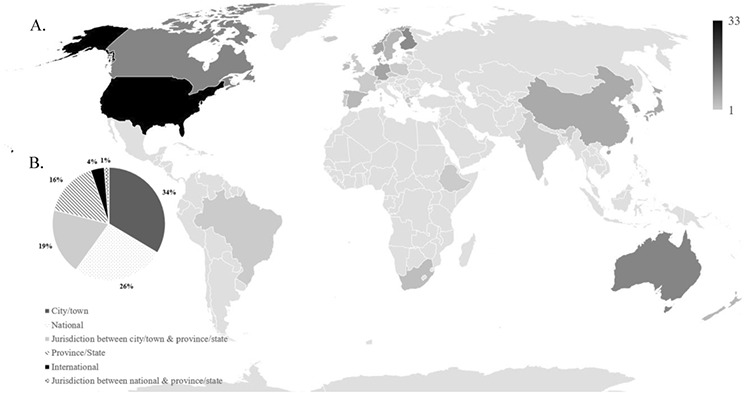

**Key Points**

- Longitudinal studies of child mental disorders investigate risk factors and sequelae of disorder.
- Comprehensive synthesis of the characteristics of existing longitudinal studies are needed to provide an understanding of studies conducted to date and to inform planning of new studies.
- This review synthesizes characteristics of 159 longitudinal studies of child mental disorders in the general population ranging in size, scope, location and duration.
- Our findings have implications regarding: (a) the usefulness of published methodology and the need for standardized reporting requirements; (b) meta‐analytic syntheses of longitudinal evidence relating to child mental disorder; and (c) planning and methodological considerations for new studies.

## INTRODUCTION

The pooled global prevalence of mental disorders among children and adolescents (herein children) has been estimated at 13.4% (Polanczyk et al., 2015), representing individual and social burden (Lim et al., 2008; Waddell et al., 2018), and posing a considerable public health concern (Kvalsvig et al., 2014). Longitudinal studies of child mental disorders in the general population have advanced our understanding of trends in prevalence and incidence of disorders; risk and protective factors; and sequelae. Early studies such as the 1946 UK National Birth Cohort, the 1981 Finnish Birth Cohort, the 1983 Ontario Child Health Study, the 1981 Queensland Study of Pregnancy, the 1975 Dunedin Multidisciplinary Health and Development Study, and the 1958 US National Collaborative Perinatal Project (or Child Growth and Development Study) identified risk factors for mental disorders in children including long or repeated hospitalizations from birth to age five (Douglas, 1975), parental separation, adoption (Ely et al., 1999; Lipman et al., 1992), disrupted familial relationships (Almqvist et al., 1999), maternal anxiety (McClure et al., 2001; Spence et al., 2002), poor cognitive functioning, parental psychopathology and early aggression (Schonfeld et al., 1988). Longitudinal research has also identified factors that mediate associations between low socio‐economic status and symptoms of disorder (Bor et al., 1997). Knowledge of risk and protective factors and sequelae related to child mental disorders expand our understanding of the development and impact of disorders and serve as targets for prevention, treatment, and intervention (Trentacosta et al., 2008). These factors occur at the individual, family, and environmental levels (Cabaj et al., 2014), have been conceptualized diversely as downstream, midstream and upstream (Dopp & Lantz, 2020) or distal and proximal (Lämmle et al., 2013), in different system and socio‐ecological models (Bronfenbrenner, 1989; Stormshak & Dishion, 2002), and are captured using different methods and informants. This complexity makes it difficult to categorize and compare risk and protective factors across studies.

Studies that recruit samples of children from the general population are the focus of our review. Our definition of general population studies follows Polanczyk et al. (2015), as studies deliberately recruiting representative samples to produce findings that are generalizable to the general population. This is achieved through probability sampling from a sampling frame that is inclusive of the target population, ensuring that everyone in the target population has a known chance of being selected. General population studies are the focus for the following reasons. First, they provide a holistic representation of mental disorders by including children across the disorder spectrum as opposed to including only the most severe, and often more complex, cases (Jongerden et al., 2015). As comorbidity is higher in clinical samples, knowledge gained from them may not fully represent the disorder in the general population (McConaughy & Achenbach, 1994). Second, general population studies include symptomatic children whose parents/teachers or who themselves do not seek care, important for comparability when the reasons for help‐seeking and access to care differ across countries and populations (Georgiades et al., 2019; Costello et al., 2014; Hintzpeter et al., 2015; ten Have et al., 2009; Fekih‐Romdhane et al., 2022). Children exhibiting externalizing behaviours may be more commonly referred when help‐seeking is largely driven by parents and/or teachers who can observe externalizing behaviours more easily or perceive them as more of a nuisance (De Los Reyes et al., 2015; Splett et al., 2019). In addition, there may be social, financial and/or geographical barriers to help‐seeking, such as living in places where beliefs that mental disorders do not need treatment are common, or where mental illnesses are not considered as urgent or dangerous as physical illnesses, and so not a resourcing priority (O’Brien et al., 2016; Radez et al., 2021). Third, general population studies produce the most widely generalizable results—making them useful for informing public funding and policy decisions at the population level.

The Diagnostic and Statistical Manual of Mental Disorders (5th ed.; DSM‐5; American Psychiatric Association, 2013) and the International Statistical Classification of Diseases and Related Health Problems (11th ed.; ICD‐11; World Health Organization [WHO], 2019) are common classification systems of mental disorders but have limitations (Doernberg & Hollander, 2016; Sleep et al., 2021). In this review, we define mental disorders broadly as “a clinically recognizable set of symptoms or behaviours associated in most cases with distress and with interference with personal functions” (WHO p.11, 1992) to accommodate variability in conceptualizations. We focus on assessment of symptoms to accommodate the multiple measurement tools and methods developed in accordance with different classification frameworks and do not require conjoint assessment of impairment. Disorder selection is broad and includes disorders commonly reported in children such as depressive, anxiety, attention, affective, oppositional defiant, conduct, eating, and substance use disorders (Polanczyk et al., 2015). We target studies using valid and reliable disorder measurement methods such as standardized diagnostic interviews or self‐completed symptom checklists (Boyle et al., 2017). Assessments can be completed by multiple informants as they provide context‐dependent information about disorder manifestation (De Los Reyes et al., 2015). We target studies that use at least a parent or child informant. Parents are the preferred informant for younger children until they become old enough to adequately recognize and report their own symptoms (Smith, 2007), usually around age 12. Teachers may also report on a child's disorder symptoms, particularly for older children and externalizing symptoms.

Longitudinal studies of child mental disorders in the general population are crucial for examining temporal trends, supporting causal inference, characterizing unique developmental trajectories, and understanding risk and protective factors that influence outcomes later in a child's life (Holz et al., 2021). Compared to cross‐sectional studies, the design features of longitudinal studies enable: (1) temporal analyses that identify age of onset and duration of mental disorders (Tejerina‐Arreal et al., 2020); (2) identification of trajectories of disorder, including periods of improvement, deterioration, and stability (Farrington, 1991); (3) opportunities for inference about effects of intervention or exposure, which may serve as targets for prevention strategies (Lockhart et al., 2018; Miller et al., 2018; Suveg et al., 2011); and (4) the separation of age, period and cohort effects in observed trends (Diggle et al., 2002). Still, the advantages of longitudinal studies must be weighed against their time and resource intensity. They require continual securing of funding, planning and commitment from researchers and participants (Farrington, 1991). Large research teams are needed, and members may have different interests (Grammer et al., 2013). Substantial efforts are also necessary to prevent attrition and maintain external validity (Fröjd et al., 2011; Gustavson et al., 2012). Longitudinal studies of child mental disorders in the general population must be carefully and deliberately planned to maximize utility and ensure efficient use of resources. Given research investments to date, it is important to understand what longitudinal studies of child mental disorders have been conducted and their methodology, objectives, and operational characteristics, so that new studies can align with existing methodology or add novel features. In this review, these characteristics are grouped as follows: the study operational factors, temporality, sampling and sample attributes, mental disorder measurement, and the information sources and content (risk and protective factors, and sequelae) assessed. This information can motivate the development of new research questions using these studies, the synthesis of evidence across multiple studies and can inform the planning of new longitudinal studies.

### Operational factors

These include *study objectives*, *funding* *sources*, and *geographical location*, as they have the potential to influence study methods. *Data accessibility* is a characteristic of interest based on the continued open data and sharing efforts in the scientific community to increase research efficiency, transparency, and replicability of published results (Mello et al., 2013). Better data access maximizes study value, potential output and removes cost and access barriers to obtaining longitudinal data, but also comes with logistical and resourcing challenges. Text about *unique study features* was also extracted.

### Temporal characteristics

These include the *year of study initiation, year of most recent follow‐up*, *number of follow‐ups*, and *whether studies are ongoing* which provide context on the objectives and study progression. A greater number of follow‐ups may improve precision when assessing disorder patterns over time (Willett et al., 1998) but may also increase attrition due to increased burden on respondents. *Study duration* is important as longer duration allows for inclusion of more distal risk factors including inter‐generational relationships (Trentacosta et al., 2008). *Age of the sample* places the study into a developmental context as disorder trajectories can be age‐dependent (Holmbeck et al., 2006; Oerlemans et al., 2020).

### Sampling and sample attributes

These include *target population, sampling frame,* and *sampling approach*. For children, appropriate sampling frames include birth records and registries, child tax benefit records, and household censuses. Schools may also be acceptable sampling frames in areas where practically all children attend (Polanczyk et al., 2015). Achieving high *response rates* further increases the probability that a sample is generalizable to the target population, in this case the general population (Weitzman et al., 2003). Researchers may also check the representativeness of their sample by comparing demographic factors such as sex between their sample and target population or by using sampling weights (Schulz et al., 2021). Attrition is a potential source of bias in longitudinal research and a major concern, with evidence indicating special populations (children with more severe symptoms, ethnic/racial minorities, and males) are more likely than their counterparts to be lost to follow‐up (Gustavson et al., 2012). The *use of incentives or gifts to participants* has been shown to reduce attrition (Booker et al., 2011).

Researchers seeking to study rare disorders may *oversample* high risk individuals and special groups to increase the statistical power of their study and maximize resources (de la Osa et al., 2019; McGonagle & Sastry, 2015). Similarly, funding challenges may require investigators to create *subsamples* of general population studies (Carter et al., 2010). *Pregnancy cohorts*, or studies recruiting pregnant women and initiating data collection before children are born, do not target children as their study population. Nonetheless, certain prenatal and perinatal circumstances have been associated with increased risk of mental disorders later in childhood (Allen et al., 1998; Ståhlberg et al., 2020) and are included in our review if they meet inclusion criteria.

### Mental disorder measurement

This includes the *name* and *type of assessment tool* (standardized diagnostic interview, dimensional measure, researcher developed measure), *disorder coverage* (neuro‐developmental or cognitive; psychotic and dissociative; mood or anxiety; obsessive compulsive; trauma and stressor‐related; somatic; eating, elimination, or sleep; sex‐related; gender dysphoria; disruptive, impulse control, or conduct; personality; and substance use and other addictive disorders in childhood) and *informant*.

### Information sources and content

We identified *data sources, content* and *informants*. Categories of study content were informed by our understanding of key risk and protective factors and are organized by *collection mode*. Data sources include: (1) *data linkage* to administrative health or government records relating to service use, medication use, or education which provides access to more detailed information with better reliability, less bias, and in a way that can reduce respondent burden (Harron et al., 2017); (2) *ecological momentary assessment (EMA)* which involves continual assessment of study participants in their natural environments using participant‐reports or sensors increasing ecological validity, reducing recall bias and random error, and in some instances increasing sensitivity to change (Moskowitz & Young, 2006); (3) *observational assessments*, where study participants' behaviors are observed for specified characteristics while engaged in a task assessing study participants' interactions with their environment and other people (Floyd et al., 1998); and (4) *biological samples* which increase our understanding of biological determinants, progression and sequelae, and the genetic basis underlying psychopathology (Insel et al., 2010).

### Objectives

We conducted a systematic review of the study characteristics (operational factors, temporality, sampling and sample, mental disorder measurement, and information sources and content) of longitudinal studies of child mental disorders in the general population to describe the range, scope, and nature of studies conducted to date. A search of JBI Evidence Synthesis, the Cochrane Database of Systematic Reviews, PubMed, Epistemonikos figshare, OSF, and PROSPERO found no reviews of all available general population longitudinal studies that measure mental disorders in children and map risk and protective factors across a broad age span. With the large number of longitudinal studies conducted, this information is needed to: (1) understand which studies have been conducted to date; (2) provide a study inventory for researchers to identify evidence for comparison, harmonization, replication, meta‐analysis, or application of machine learning approaches; and (3) inform the deliberate and strategic planning of new longitudinal studies to maximize the value of research investments. Bringing existing longitudinal evidence to bear on new research questions can consolidate cross‐study findings and identify sources of stability or change in trends and associations (Ioannidis & Lau, 1999).

## METHODS

### Protocol and registration

Study methods adhered to Cochrane (Higgins & Green, 2011) and the Preferred Reporting Items for Systematic Reviews and Meta‐Analyses (PRISMA: Moher et al., 2009) standards for systematic reviews. The review was registered with Open Science Framework (OSF) (registration DOI: 10.17605/OSF.IO/73HSW) and all data is available through OSF (https://osf.io/gzm58/).

### Literature search

A systematic literature search with no date limits was conducted first in July 2021 and repeated in December 2022 to update the review using MEDLINE, EMBASE and PsycINFO via the Ovid platform. The search strategy (Appendix 1) was developed in consultation with an experienced health science librarian (LB). Based on our inclusion/exclusion criteria (Appendix 2), a search of MEDLINE was conducted first. Titles, abstracts, and index terms were reviewed for relevant terms which were added to the search strategy. The final search strategy was reproduced in EMBASE and PsycINFO and combined keyword and controlled vocabulary terms for: (1) the population (children from neonatal‐18); (2) study type (all types of longitudinal study); (3) common mental disorder types (depressive, anxiety, attention, affective, oppositional defiant, conduct, eating, and substance use disorders, and groupings of mental, emotional, mood, behavioural, internalizing and externalizing); and (4) record type (epidemiological, methodological, or prevalence).

### Study identification and eligibility

The target of the review is the *study*—the operational process of designing and conducting a primary research project, collecting, and reporting data—rather than an individual *record—*a paper, manuscript, or publication. Although *study* is commonly used to refer to a published manuscript, in this review it refers to the larger scale research endeavor such as the National Longitudinal Survey of Youth, or the Millennium Cohort Study. The search strategy was developed to identify records documenting methodology, mental disorder prevalence or epidemiological research from longitudinal studies. These records were used to identify studies, screen for eligibility, and identify an appropriate information source about the study for data extraction. These could be: (1) a record documenting the study methodology; (2) a study website; (3) a study data user guide; and (4) a record documenting disorder prevalence. The research team determined source types based on those that are typically available and used to find out about study methodology. They were prioritized according to reliability and comprehensiveness (in order shown above). Having one consistent source of information would have been preferred, but no standard study reporting method exists.

Records identified by our search were uploaded into EndNote (version 20.1) and duplicates were removed. One researcher (TB) independently reviewed the titles and abstracts of retrieved records and grouped them using study name into records that included a name and records that did not. When a study name was found, all records referencing that study name were searched for and a group of records for that study was created in EndNote. A methodology paper was then searched for. If unavailable, a Google search of the study name was conducted to identify a study website or data user guide. If unavailable, the record group for a particular study was searched to find a paper documenting the prevalence of mental disorders. The study was screened for eligibility using the first available source and criteria listed in Appendix 2. Records without a study name were screened individually according to our eligibility criteria and, where possible, an information source was identified. In cases when eligibility was unclear, articles were reviewed and discussed with a senior author (LD). Eligible studies went through full text review for inclusion, in duplicate, by four trained reviewers (TB, WX, HM, BV), using one of the four specified sources, following inclusion/exclusion criteria. Discrepancies in the identification of eligible studies were resolved through discussion. When the study was potentially eligible for inclusion, but a study name was unavailable, the authors were contacted to obtain the name and an information source.

### Data extraction

Data was extracted and coded independently by four reviewers in duplicate (TB, WX, HT, HM) using a Microsoft Excel data extraction tool and codebook (Appendix 3). Reviewers were trained and a pilot extraction study (*n* = 18) was conducted on randomly selected studies by two reviewers (TB, WX). Reviewers identified, discussed, and resolved disagreements to achieve consensus and the data extraction tool was revised to add/remove fields based on rates of available data. Inter‐rater agreement on coded data was calculated as a percentage.

### Analytic approach

Data for included studies were entered into a database. Text was extracted on unique study features, target population and mental disorder measurement tools—grouped based on the tool name, taking into consideration different spellings, and abbreviations. Respondent and age‐group version information for tools was not extracted. Tabular descriptive statistics were generated in Excel and narrative syntheses were generated for extracted text.

## RESULTS

### Study selection

**FIGURE 1:**
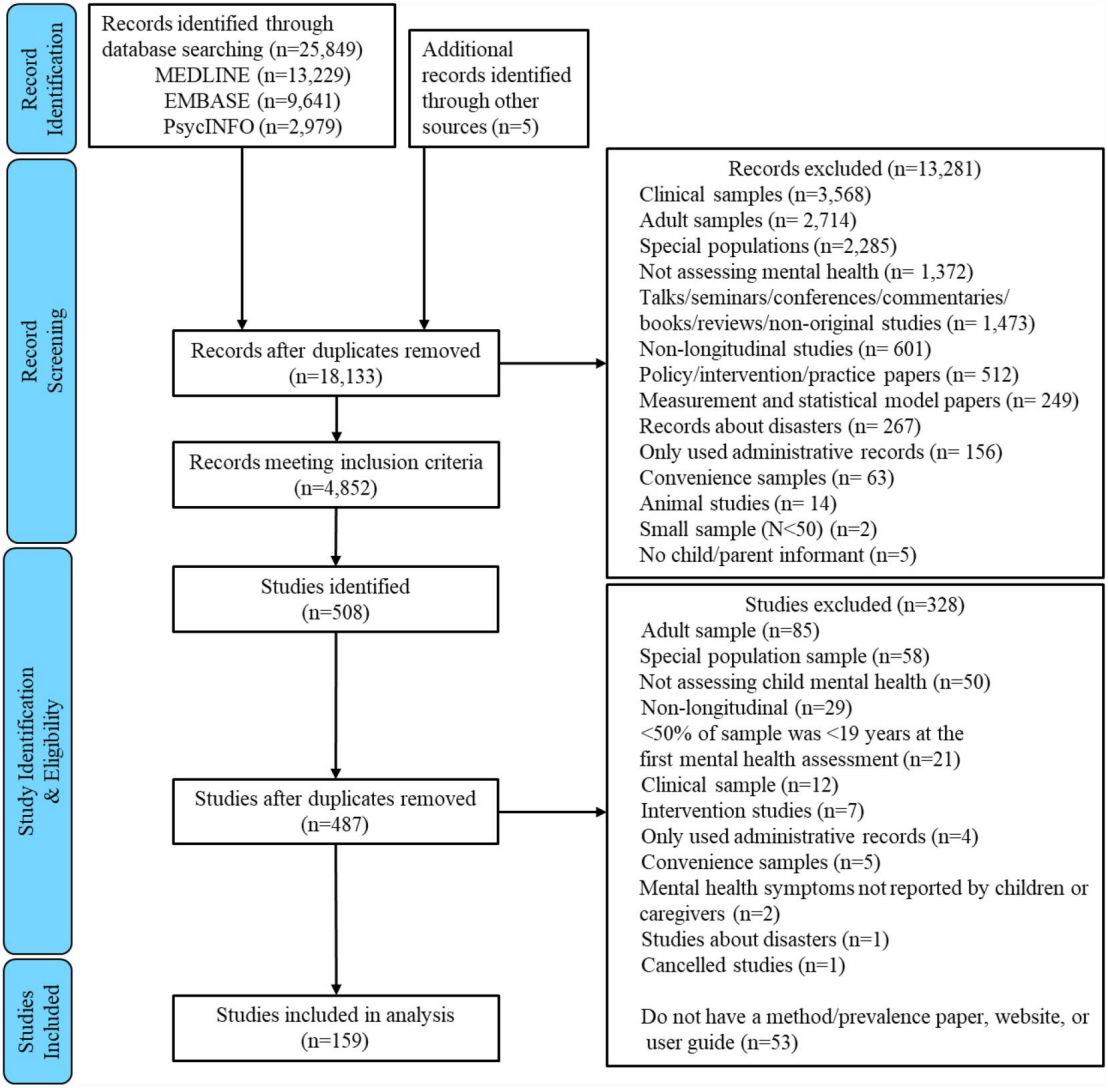
PRISMA flow diagram of study identification.

Our search strategy resulted in 18,133 unique records of which 4852 met our inclusion criteria. Of these, 3735 represented groups of records for 487 named studies and 1117 related to nameless studies. From these, 11 records reporting methods or prevalence were identified, and nine authors were contacted for study name information and a data extraction source. In total, 487 studies were identified. Figure 1 presents the PRISMA diagram for study identification. Following eligibility screening, 159 studies were included. Appendix 4 lists the eligible studies and link or citation for the information source. Initial agreement on eligibility between screeners was 88.7%, with 100% consensus achieved after discussing disagreements. Initial agreement for the extracted, coded, study characteristics was 90.3%, and 100% after disagreements were resolved.

### Operational factors

Of the 159 included studies, 94% had an identifiable study name (Table 1). Over half (54%) had a peer‐reviewed methodology paper available, which was used to assess eligibility and for data extraction. A quarter (25%) had a study website, 17% had a published mental health‐related prevalence paper, and a study user guide was used for the remaining studies (5%). Only 39% of studies reported their data accessibility. Among those that did, most required external researchers to apply for data access and/or pay a modest fee. A third had restricted data access, with access limited to collaborators; only 2% reported having some portion of study data available through open access.

**TABLE 1:** Operational, temporal, and sample study characteristics.

Characteristics	% (*N*)		Median	Range	Unreported	
					% (*N*)	
Operational factors						
Total	100%	(159)				
Had a study name	94%	(149)			6%	(10)
Data access					39%	(62)
Gated	65%	(63)				
Restricted	34%	(33)				
Open	2%	(2)				
Temporal characteristics						
Start year			2003	1934–2019	6%	(10)
End year			2014	1975–2022	19%	(30)
Duration (years)			10	0–83	19%	(30)
Follow‐ups to date			4	1–68	11%	(18)
Ongoing	70%	(97)			13%	21
Sample characteristics						
Stated target population	87%	(138)			N/A	
Birth cohort	24%	(38)			N/A	
Baseline sample size			2792	151‐64,136	3%	(5)
Baseline response rate			84%	17%–100%	26%	(42)
Baseline sex: male			51%	28%–70%	50%	(80)
Age			8.5 years	0–24 years	2%	(3)
Sample size at final follow‐up			1699	101‐57,865	26%	(42)
Sampling approach					20%	(32)
Cluster	43%	(54)				
Total population	26%	(33)				
Stratified	25%	(32)				
Multiple	11%	(14)				
Random	9%	(12)				
Sampling frame					30%	(44)
School lists	45%	(52)				
Maternal/child health records	19%	(22)				
Multiple	8%	(9)				
Birth records	7%	(8)				
Dwelling registries	7%	(8)				
Samples from other studies	6%	(7)				
Census records/population registry	4%	(5)				
Child benefit records	2%	(2)				
Phone directories	1%	(1)				
Other registries	1%	(1)				
Oversampled special groups	26%	(36)			14%	23
Incentives used	26%	(32)			21%	34
Unique study feature	30%	(43)				18

**FIGURE 2:**
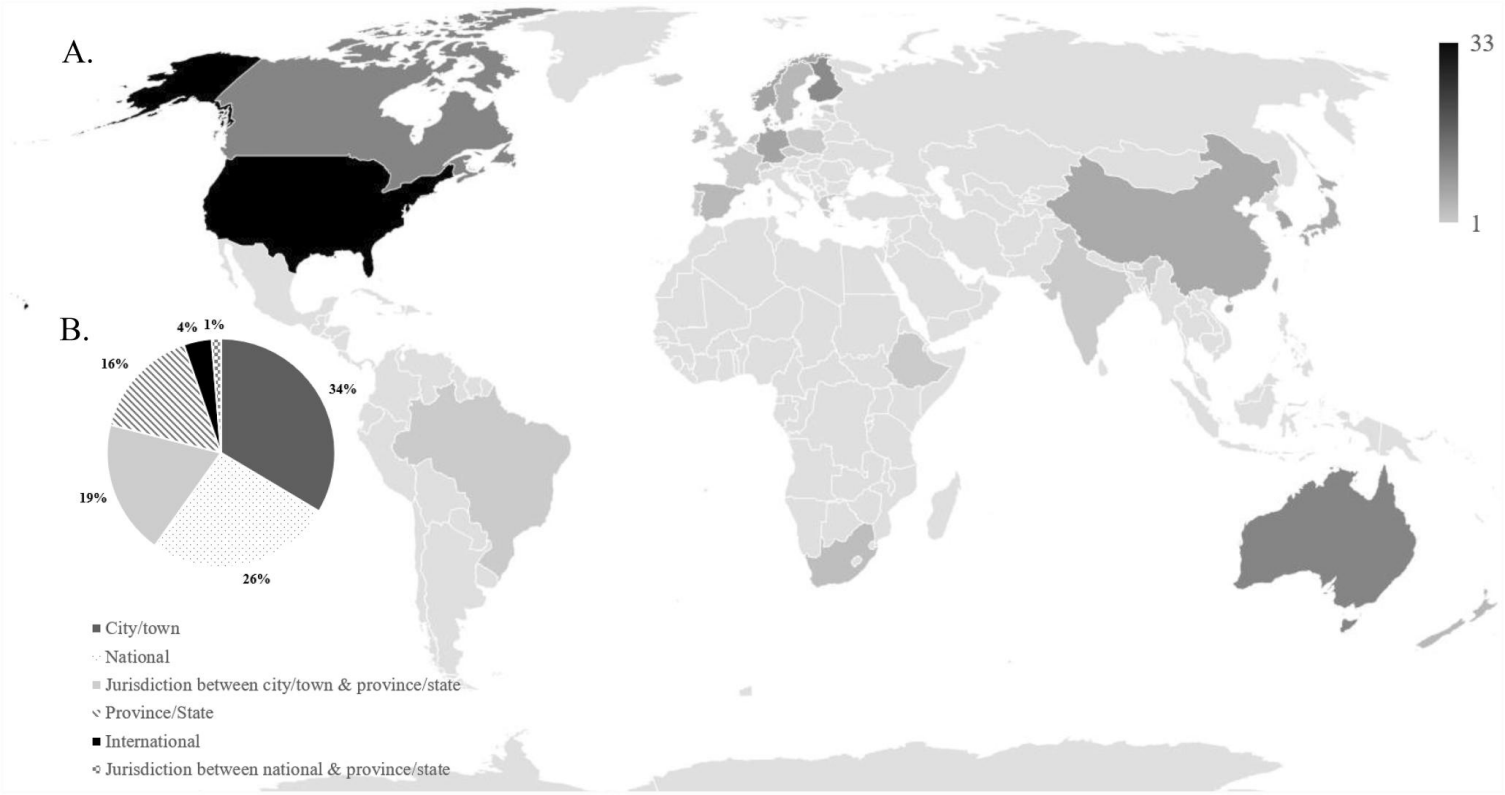
Geographical locations (A) and scope (B) of eligible studies.

Studies were conducted in all continents (Figure 2). At the country level, most studies were conducted in the United States (20%), followed by England (8%), Australia (7%), and Canada (7%). One study did not specify a geographic region. Studies were most often conducted at the town/city level (34%), followed by the national level (26%) and a jurisdiction between city/town and state/province (19%). Studies were less frequently conducted at the province/state (16%), international level (4%) or at a jurisdiction between national and provincial (1%).

Forty‐three studies reported a unique design feature. The majority (*n* = 20) described a connection or linkage to another study that emerged out of the original study or allowed access to data on other family members, laboratory studies, or similar (‘consortium’) studies in other countries. Eleven described a subsample or some aspect of special sample targeting conducted as part of the study. Six studies noted something unique about their approach to measurement (e.g., sipping/tasting vs. drinking alcohol, fathers as respondents, sexual behaviours questions adapted for Muslim population, mental disorder case definitions). Special foci on system influences, large size, unique characteristics of study location, and the ability to use non‐participants as controls were also described.

### Temporal characteristics (Table 1)

Year of study initiation ranged from 1934 to 2019, with the median reported study duration and follow‐up number being 10 years and four follow‐ups. Seventy percent of the studies were ongoing. Attrition, perhaps the primary challenge to generalizability in longitudinal studies, was not well reported. Using baseline sample size and sample size at last reported follow‐up, we were able to calculate attrition for 73% of studies. For 5% of these, baseline sample size was smaller than follow‐up due to sample augmentation or misreporting. Median attrition rates were 30% and post hoc analysis found a median rate of 22% in studies that reported using incentives.

### Sampling & sample attributes (Table 1)

The majority (87%) of studies had a stated target population. A quarter were pregnancy cohorts. Baseline sample sizes ranged from *n* = 151 to 64,136 children (median *n* = 2792). Of the 74% of studies reporting baseline response rates, participation ranged from 17% to 100% (median = 84%). The median age for study participants was 8.5 years. Half (50%) did not report study participants' sex at baseline. For those that did, the median proportion of males was 51%.

Of the 127 studies that reported sampling approaches (80%), cluster sampling was reported most (43%), followed by total population and stratified sampling (26%), often in combination with other sampling approaches. Sampling frames were reported by 70% of studies. Over half of studies used school lists and a fifth used maternal and/or child health records. Studies also reported using birth records, dwelling registries, samples from other studies or multiple frames. Less commonly used sampling frames were child benefit records and phone registries. A quarter of the studies reported oversampling high risk or special populations of interests for their studies and providing incentives to their participants.

### Mental disorder measurement

Studies assessed children for mental disorder symptoms using various instruments and informants (Table 2). Internalizing disorder symptoms were assessed most, followed by disruptive, impulse control and conduct, neurodevelopmental and neurocognitive, and eating, elimination, and sleep. Some studies assessed children for substance use and other addictive disorder symptoms, somatic, psychotic and dissociative, obsessive compulsive, and trauma/stressor‐related disorder symptoms. Few studies reported measuring personality disorder, and gender dysphoria symptoms.

**TABLE 2:** Mental disorder assessment characteristics.

	% (*N*)		Unreported	
			% (*N*)	
Disorder Class			6%	(10)
Internalizing	78%	(116)		
Disruptive, impulse control, conduct	61%	(90)		
Neuro‐developmental/cognitive	60%	(89)		
Eating, elimination, sleep	40%	(59)		
Substance use & other addictive	33%	(49)		
Somatic	12%	(18)		
Psychotic & dissociative	11%	(17)		
Obsessive compulsive	10%	(15)		
Trauma/stressor‐related	5%	(8)		
Personality	3%	(4)		
Gender dysphoria	1%	(1)		
Sex‐related	0%	(0)		
Instrument			25%	(39)
Measurement scale	91%	(109)		
Standardized diagnostic interview	28%	(33)		
Researcher developed	18%	(22)		
Commonly Used Assessments			29%	(46)
CBCL (scale)	33%	(37)		
SDQ (scale)	30%	(34)		
CES‐D (interview)	10%	(11)		
MFQ (scale)	10%	(10)		
YSR (scale)	9%	(10)		
DISC (interview)	8%	(9)		
K‐SADS (interview)	7%	(8)		
CDI (interview)	6%	(7)		
Informants			1%	(2)
Child	67%	(105)		
Caregiver	59%	(85)		
Teacher	22%	(34)		
Service provider	2%	(3)		

Abbreviations: CBCL, Child Behaviour Checklist; CDI, Children’s Depression Inventory; CES‐D, Center for Epidemiologic Studies Depression Scale; DISC, Diagnostic Interview Schedule for Children; K‐SADS, Kiddie Schedule for Affective Disorders and Schizophrenia; MFQ, Mood and Feelings Questionnaire; SDQ, Strengths and Difficulties Questionnaire; YSR, Youth Self‐Report.

Measurement scales were used in almost all studies—far more frequently than diagnostic interviews; a quarter of studies used both assessment types. Researcher‐developed instruments were also used. Commonly used measurement scales include the Child Behaviour Checklist (CBCL; Achenbach, 1999), Strengths and Difficulties Questionnaire (SDQ; Goodman, 1999), Mood and Feelings Questionnaire (MFQ; Angold et al., 1995), and Center for Epidemiologic Studies Depression Scale (CES‐D; Radloff, 1977), Children's Depression Inventory (CDI; Saylor et al., 1984), and Youth Self Report (YSR; Ebesutani et al., 2011). The most frequently reported diagnostic interviews were the Kiddie Schedule for Affective Disorders and Schizophrenia (K‐SADS; Kaufman et al., 1997) and Diagnostic Interview of Children and Adolescents (DISC; Shaffer et al., 2000). Most studies used child or caregiver reports of the child's mental disorder symptoms, while fewer studies also included teacher and service provider reports.

### Information sources and content

Information sources and types of information collected were coded as present if reported and assumed to be absent if not reported (Table 3). All studies reported at least one information type and 5 did not report a source. In addition to reports from multiple informants about child mental disorders, studies collected information from other sources. These included data from linkages to school or medical records, neurocognitive testing, or biological samples most commonly. Some studies included an observational assessment of the child, EMA assessment data, such as written or electronic diaries, app‐based data, or physiological sensors, or interviewer assessments of the child, their family, or environment.

**TABLE 3:** Information sources and content characteristics.

	%	(*N*)	Unreported	
			% (*N*)	
Sources			3%	(5)
Administrative individual data linkage	47%	(73)		
Neurocognitive testing	44%	(67)		
Biological‐other	37%	(57)		
Biological‐genetic	34%	(53)		
Observational	18%	(27)		
Ecological Momentary Assessment (EMA)	12%	(19)		
Interviewer‐rated content	9%	(14)		
Content Assessed			0%	(0)
Environmental factors				
Neighbourhood/Community	43%	(69)		
Familial factors				
Socio‐demographic/‐economic factors	94%	(150)		
Parenting	76%	(121)		
Parental mental health & substance use	62%	(99)		
Parental physical health	50%	(79)		
Family functioning	45%	(71)		
Abuse, trauma, extreme adversity	21%	(34)		
Individual factors				
Physical health	81%	(128)		
Lifestyle	77%	(122)		
School	69%	(110)		
Psychological	68%	(108)		
Birth history/development	64%	(101)		
Service Use	59%	(94)		
Peer relationships	54%	(86)		
Adverse Childhood Experiences (ACEs)	43%	(69)		

Risk and protective factors and/or sequelae of mental disorders were grouped according to whether their content was about the individual, family, or broader environment. At the individual level, studies collected information about the child's physical health, lifestyle, school and academics, psychology (i.e., temperament personality, self esteem), birth history and physical development, service use (including community, emergency, or specialized medical services), peer relationships, and adverse childhood experiences. Almost two thirds of studies reported assessing service use, including community, emergency, or specialized medical services. Commonly assessed familial factors include socio‐economic and ‐demographic factors, parenting and family structure, parental mental health and/or substance use, parental physical health, family functioning, and extreme parental adversity. At the environmental level, half of studies reported collecting information on study participants' neighbourhood and community.

## DISCUSSION

This review describes and synthesizes the reported methodological features of longitudinal studies of child mental disorders providing a comprehensive understanding of the characteristics of studies that have been conducted to date. The studies identified use diverse methodologies, are conducted worldwide, and represent a broad span of time over which studies of varying duration were conducted, and for the majority, continue to be conducted. The high proportion of ongoing studies underscores the value of longitudinal research in academia, the potential for generating meta‐analytic research questions, and interest in studying intergenerational risk and protective factors, sequelae, and factors that are more temporally distal. This review identifies common approaches to sampling and measurement and presents recommendations and findings that can be used to inform the planning of new longitudinal studies and identify areas for methodological innovation in future studies (Table 4).

**TABLE 4:** Common methodology of existing studies and general recommendations for longitudinal studies of child mental disorder.

Common existing longitudinal study methodology	
A. Operational	Target geography from town/city to national level
B. Temporal	Median 10‐year duration with data collection at 5 time points
C. Sampling and Sample	Cluster or total population sampling
	School lists as sampling frame
	Baseline sample size of ∼*n* = 2800
D. Mental Disorder Measurement	Disorder inclusions by frequency of measurement: internalizing, externalizing, neuro‐developmental, eating/elimination/sleep, substance use/addictive
	Use of measurement scales to assess disorder
	Use of child and parent/caregiver informants, some also include teacher
E. Information Sources and Content	Common additional data sources: linkage to school or medical administrative data records, neurocognitive tests, biological samples to assess genetic or other characteristics
	Common content by frequency of collection Individual: child physical health, lifestyle, school, psychological, birth history and development, service use, peer relationships, adverse childhood experiences Familial: socio‐economic/‐demographic factors, parenting and family structure, parental mental health and/or substance use, parental physical health, family functioning, parental adversity Environmental: neighbourhood and community characteristics

General recommendations	
1. Report study methodology according to existing guidelines/checklists; a published study methodology/protocol paper is recommended	
2. Use a standard study name across publications to assist with future identification of studies	
3. Assess completeness of sampling frame for representing general population	
4. Report data accessibility and process for access	
5. Report response and retention rates	
6. Use incentives to reduce attrition	
7. Consider opportunities to connect or link to other existing studies, embed in or add to an existing cohort, or join a study consortium	

This review serves as an inventory of studies conducted to date and the supplementary data file of extracted characteristics can be used to find studies as sources of evidence for comparing or replicating results, harmonizing new study methodology with existing studies, identifying possibilities for conducting meta‐analysis, or finding sources of ‘big data’ for applying machine learning approaches that need large amounts of data on different variables, individuals and time points (Dwyer & Koutsouleris, 2022). Our review suggests comparison or meta‐analysis across cohorts would be possible. Meta‐analysis is especially viable given that almost all studies used measurement scales to assess children for mental disorder symptoms and most studies assessed similar disorder types. Study inclusion should not require the use of the same measurement scale given that the most common measure, the CBCL, was only used in around a third of studies.

Not all researchers conducting longitudinal studies publish their study methodology. For publications using longitudinal data, there is no common reporting methodology or consensus on what should be reported. While reporting guidelines have been developed and their use is increasing (e.g. Strengthening the Reporting of Observational Studies in Epidemiology (STROBE; Vandenbroucke et al., 2007); Reporting of studies Conducted using Observational Routinely‐collected health Data (RECORD; Benchimol et al., 2015); Transparent reporting of a multivariable prediction model for individual prognosis or diagnosis (TRIPOD; Collins et al., 2015)), journals are inconsistent in requiring their use. This represents a challenge to information synthesis. To overcome this challenge, we identified four potential information sources to increase the likelihood of being able to access data for extraction. We clearly report where information was missing for studies and interpret results for those studies that did report information. We encourage researchers conducting or publishing longitudinal studies to use reporting guidelines or publish a study methodology paper or cohort profile such as those published in the International Journal of Epidemiology (https://academic.oup.com/ije/search‐results?f_TocHeadingTitle=Cohort+profiles&sort=Date+%e2%80%93+Newest+First). A compendium of longitudinal studies would be a useful resource.

There is an increasing expectation for researchers to share the data they report on (Mello et al., 2013) although sharing also raises concerns around informed consent, data management, data dissemination, and validation of research contributions (Alter & Vardigan, 2015) and has logistical and cost implications (Hamilton et al., 2023). Many studies did not report on data accessibility and of those that did, the majority reported restricted access—mainly due to data privacy—or required application and/or payment for data access. It's not clear if these fees are aligned with the costs incurred from sharing (e.g. data extraction, cleaning, drafting agreements, securing data transfers, follow‐up on research contributions). Post hoc analysis exploring whether researchers are making their data available in response to these expectations found that accessibility of data was not becoming more common over time, which suggests that challenges to openly accessible data have not yet been resolved. Previous studies have also documented potential reluctance among researchers to share raw data which could also be a contributing factor (Stieglitz et al., 2020; Zhu, 2020). Open data access increases transparency by allowing other researchers to verify research, gives general access to publicly funded research and cost‐effectively advances research (Borgman, 2012). However, it is not clear whose responsibility it is to develop sharing processes, navigate privacy laws and cover accompanying costs, factors that are further complicated when sharing crosses national borders.

School lists were the most popular sampling frame but come with limitations that should be considered and assessed, if possible. School lists work when virtually all children attend (Polanczyk et al., 2015), but it's unclear if or how this was assessed when school list sampling frames were used. Globally, 87% of children attend primary school, rates differ by location, and education level (United Nations International Children's Emergency Fund (UNICEF), 2022). Low income at the country level and individual level are associated with lower school attendance rates (Sosu et al., 2021). It is possible that studies sampling from school lists are missing individuals and groups that are at higher risk for mental disorders (Dupéré et al., 2018; Hjorth et al., 2016), thereby reducing the generalizability of findings. This limitation is also a concern for studies of secondary school children, where school drop‐out occurs more frequently (Gubbels et al., 2019) and respondents tend to be youth, rather than their parents, adding complexities around consent, privacy and respondent motivation (Bonnell et al., 2018). Administrative maternal and child medical records were also frequently used. Health records may not lead to the recruitment of representative general population samples when women forgo services or in countries with high rates of immigrant families with children born in another country. Birth records also have limitations due to omission of immigrant and refugee children. A completeness assessment of commonly used sampling frames or identification of alternate, more complete sampling frames (e.g., using census or administrative tax file data to identify families with children) would preserve and potentially enhance the applicability of results from general population studies.

Sampling approaches varied, but as school‐based samples are largely cluster samples, this was the most common approach, often in combination with stratification or random sampling. Cluster sampling can increase efficiency in large‐scale studies (Latpate et al., 2021), but the representativeness of samples produced using this method can be challenged when many clusters (i.e., schools) refuse participation. While total population sampling, the second most frequent method, improves representativeness of the sample, it is expensive, and is still subject to nonresponse. General population study response rates have declined over time (Ebert et al., 2018; Stedman et al., 2019). Response rates in this review had a wide range, suggesting that study response remains a challenge and researchers should continue to employ strategies to encourage response, particularly strategies focused on reducing barriers to participation, such as the provision of childcare, transportation, and parking services. The more important issue in longitudinal studies is retention of survey respondents over time and the bias that can result from attrition. In addition to monitoring and reporting retention rates, methods exist to reduce attrition (Abshire et al., 2017), such as use of incentives, and attrition can be accounted for in data analysis using suitable missing data methods or statistical adjustments (Enders, 2011; Nicholson et al., 2016; Schminkey et al., 2016). Retention rates were not well reported and, after piloting data extraction, were removed as a field for charting due to missing data. Sample size at baseline and final reported follow‐up was reported allowing for sample retention calculations, but it is impossible to know how well this approximates actual retention. Longitudinal studies should monitor and report retention, detail any sample supplements and methodological or statistical efforts to account for sample loss.

The database search was limited to English language records for feasibility and resource reasons (costs of translators/translations), excluding otherwise eligible studies that did not publish records in English and leading to potential bias in our understanding (Amano et al., 2016). According to our review, the countries with the most longitudinal studies of child mental disorder are the United States, England, Canada and Australia. To understand the impact of omitting studies without English records, the excluded non‐English search results are reported in Appendix 5. In our search, 886 out of 18,133 records were identified as non‐English. The English titles and abstracts were screened and 94 identified as potentially eligible, corresponding to 57 studies. Of these, 23 also had English records and had already been captured by our review. Appendix 5 lists the 34 potentially eligible non‐English studies. They include records in Chinese, German, Spanish, French, Japanese, Polish, Portuguese, Italian, and Russian, reporting studies from China (8), Germany (3), Spain (3), Japan (3), France (2), Brazil (2), Italy (1), Canada (1), Chile (1), and Taiwan (1) and studies with no country reported (9). If these studies proved eligible for inclusion following full text review, this wouldn't change the finding that most studies were conducted in the United States (*n* = 33) but the representation of studies from China would increase, with China and England both having 14 studies, followed by Canada with 13. English records reporting 6 Chinese studies were included in our review. Apart from the underrepresentation of studies from China, we don't think that including non‐English language records would have led to significantly different results. Our Appendix Table A1 reports where possible, the start year, end year, location, baseline sample size and age at baseline so the reader can judge for themselves.

With or without potentially eligible non‐English studies, longitudinal studies were conducted in all continents, with European and North American being the most common and South American and African countries being the least common. This may have implications for meta‐analytic research about longitudinal relationships between mental disorders and other variables at a global level, as we know that there are cultural differences in mental disorder perceptions, mental health literacy, and help‐seeking behaviours in children (Ivanova et al., 2015, 2022; Pescosolido, 2007). Some studies noted connections to similar studies in other countries or mentioned being part of cross‐national consortium studies. Studies were most often conducted at the city/town level, followed by the national and provincial/state level. The tendency towards a smaller geographic scope is important for meta‐analytic research given almost all studies conducted at the city/town level took place in large, urban cities. There are known differences between urban and rural settings in prevalence and determinants of child mental disorders and while cities are most likely to be considered urban, towns could be urban or rural depending on size and density (Buttazzoni et al., 2022).

Most studies sought to measure a broad range of psychopathology. Fewer studies assessed disorders seen more rarely in children. This is likely due to the fact our search criteria included terms for these more common disorders, but also because general population samples are less appropriate for assessing rare disorders because large sample sizes are needed to achieve adequate statistical power. Measurement scales were used more frequently than diagnostic interviews to assess disorder symptoms in children—some studies used both. Validated measurement scales are less burdensome on respondents and interviewers and produce valid and reliable assessments increasing feasibility (Boyle et al., 2017). Most studies used multiple informants, most commonly child and/or parent/caregiver. Informant discrepancies in reports of mental disorder symptoms are known to occur, so including multiple respondents provides a comprehensive assessment of the occurrence of mental disorders (Brown et al., 2006). How to combine multi‐informant data is not well understood but we can use multi‐informant data simultaneously in latent variables in structural equation models (Martel et al., 2017).

The use of different information sources has implications for study procedures. The frequent use of data linkage to other records and the collection of biological samples represent a challenge to data sharing as these data often need more secure storage and use than traditional survey data (Harron et al., 2017; Rychnovská, 2021). Data linkage can minimize respondent burden by providing an alternate method for assessing certain survey content. Biological sample collection varies in respondent burden, is sometimes invasive and has data storage implications (International Society for Biological and Environmental Repositories, 2008). More importantly, there is some evidence from the UK that including a nurse visit for biological data collection in longitudinal studies has a negative effect on cooperation in the wave directly after the visit, although the effect is mostly short‐term and these visits did not have a longer‐term impact on subsequent wave participation (Pashazadeh, et al., 2021). Some cognitive tests, observational and interviewer assessments require the presence of a human interviewer or assessor requiring resources beyond those needed for an online or computer‐administered survey. Ecological Momentary Assessments are well‐suited to the increasing availability, affordability and use of electronic devices, technology and software programs. They can be used to administer neurocognitive tests, however these assessments can be burdensome for participants and require substantial data monitoring from researchers to minimize the amount of missing and incorrect data. The technology associated with this form of data collection may also be expensive and require resourcing to monitor and maintain to avoid software malfunction and/or data loss (Heron et al., 2017).

Our review was limited by the reporting quality of eligible studies. While some studies had detailed records of their methodology, others did not, meaning we were unable to determine some study features. For studies where a mental disorder prevalence paper was used for data extraction, mental disorder risk and protective factors, and/or sequelae may have been measured but not reported because of their limited relevance to the focus of the paper. Using methodology papers to extract the characteristics of ongoing studies may have led us to report older study features because of the typically large time intervals between methodological publications on different study waves.

To standardize data extraction and ensure that data extraction sources contain sufficient information of interest, we selected study methodology papers, websites, data user guides or mental disorder prevalence papers as information sources. While this increased the amount and standardization of information extracted, requiring that a study have one of these sources available for it to be included in the review led to the exclusion of 53 records about nameless or otherwise unidentifiable studies which could not otherwise be excluded based on our eligibility criteria. Appendix 6 lists these records. Studies not well described, easily identifiable or without consistent use of a name in published research findings are less likely to be represented in our review. Similarly, studies that address but have not published results about child mental disorders in the scientific literature are not represented.

## CONCLUSION

Longitudinal studies of mental disorders in the general population of children vary substantially in their operational, temporal, sample, disorder assessment, information and content characteristics. They occur predominantly in European and North American countries, use school list sampling frames, and assess children for a range of internalizing and externalizing disorders—mostly using measurement scales. Risk and protective factors and sequelae are assessed most frequently at the individual level, followed by familial and then environmental. This review provides a comprehensive description and synthesis of longitudinal study characteristics, inventories existing studies, provides extracted data on their characteristics, raises questions about the impact of different study methods on efficiency, internal and external validity, and provides a foundation for future meta‐analytic work. Our work recommends standardized reporting of study methodology, makes recommendations for new longitudinal studies, and identifies common methodologies that can be used when planning future longitudinal research involving the assessment of child mental disorders in the general population.

## AUTHOR CONTRIBUTIONS

**Theodora Bogdan**: Conceptualization; data curation; formal analysis; investigation; methodology; writing—original draft; writing—review & editing. **Weiyi Xie**: Data curation; formal analysis; investigation; methodology; project administration; validation; writing—review & editing. **Habeba Talaat**: Data curation; formal analysis; writing—review & editing. **Hafsa Mir**: Data curation; formal analysis; writing—review & editing. **Bhargavi Venkataraman**: Data curation; formal analysis; writing—review & editing. **Laura E. Banfield**: Conceptualization; methodology; software; writing—review & editing. **Katholiki Georgiades**: Conceptualization; methodology; supervision; writing—review & editing. **Laura Duncan**: Conceptualization; funding acquisition; investigation; methodology; project administration; resources; software; supervision; validation; writing—review & editing.

## CONFLICT OF INTEREST STATEMENT

The authors have declared that they have no competing or potential conflicts of interest.

### OPEN RESEARCH BADGES

This article has earned Open Data and Preregistered Research Designs badges. Data and the preregistered design and analysis plan are available at https://osf.io/gzm58/ and https://osf.io/73hsw.

## Ethical considerations

No ethical approval was required for this research review.

## Figures and Tables

**FIGURE A1:**
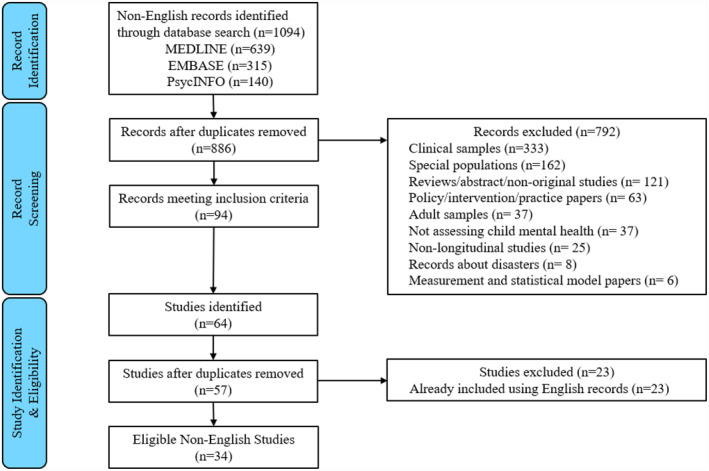
PRISMA flow diagram of study identification.

## Data Availability

The data that support the findings of this study are openly available in Open Science Framework at https://osf.io/gzm58/.

## APPENDIX 1 Search strategy

### Medline—OVID

1. mental disorders/or anxiety disorders/or anxiety, separation/or “disruptive, impulse control, and conduct disorders"/or exp “feeding and eating disorders"/or mood disorders/or depressive disorder/or depressive disorder, major/or dysthymic disorder/or neurodevelopmental disorders/or “attention deficit and disruptive behavior disorders"/or attention deficit disorder with hyperactivity/or conduct disorder/or child behavior disorders/or exp substance‐related disorders/
2. adhd. ti,ab,kf,kw.
3. addiction*.ti,ab,kf,kw.
4. ((mood or anxiety or depress* or attention‐deficit hyperactivity or oppositional‐defiant or conduct or affective or eating or “substance use”) adj3 (disorder* or condition* or symptom* or assess* or measur*)). ti,ab,kf,kw.
5. ((neuropsy* or behavio?r* or emotion* or mental* or psychiatr* or internalizing or externalizing) adj2 (diagnos* or disorder* or ill or illness*)). ti,ab,kf,kw.
6. or/1‐5
7. adolescent/or exp child/or exp infant/
8. (p?ediatric* or child* or adolescen* or youngster* or youth or teen* or boys or girls or neonat* or infan*). ti,ab,kf,kw.
9. 7 or 8
10. exp Cohort Studies/
11. ((longitudinal or follow‐up or cohort or prospective or retrospective or panel) adj2 stud*). ti,ab,kf,kw.
12. 10 or 11
13. (prevalence or survey* or epidemiolog* or methodol*). ti,ab,kf,kw.
14. Epidemiology/
15. Prevalence/
16. 13 or 14 or 15
17. 6 and 9 and 12 and 16
18. 17 not (animals/not (humans/and animals?.mp.)) [mp = title, abstract, original title, name of substance word, subject heading word, floating sub‐heading word, keyword heading word, organism supplementary concept word, protocol supplementary concept word, rare disease supplementary concept word, unique identifier, synonyms]
19. limit 18 to English
20. limit 19 to (adaptive clinical trial or address or autobiography or bibliography or biography or case reports or clinical study or clinical trial, all or clinical trial, phase i or clinical trial, phase ii or clinical trial, phase iii or clinical trial, phase iv or clinical trial, veterinary or clinical trials, veterinary as topic or clinical trial protocol or clinical trial protocols as topic or clinical trial or comment or congress or consensus development conference or consensus development conference, nih or controlled clinical trial or dataset or dictionary or directory or editorial or equivalence trial or festschrift or government publication or guideline or historical article or interactive tutorial or interview or introductory journal article or lecture or legal case or legislation or letter or news or newspaper article or observational study, veterinary or patient education handout or periodical index or personal narrative or portrait or practice guideline or pragmatic clinical trial or randomized controlled trial or randomized controlled trial, veterinary or technical report or twin study or video‐audio media or webcast)
21. 19 not 20

### PsycINFO—OVID

1. anxiety disorders/or generalized anxiety disorder/or separation anxiety disorder/
2. exp eating disorders/
3. major depression/or dysthymic disorder/or endogenous depression/or reactive depression/
4. disruptive behavior disorders/or conduct disorder/or oppositional defiant disorder/
5. attention deficit disorder/or attention deficit disorder with hyperactivity/
6. exp “substance use disorder”/
7. social anxiety/
8. adhd. ti,ab.
9. addiction*.ti,ab.
10. ((mood or anxiety or depress* or attention‐deficit hyperactivity or oppositional‐defiant or conduct or affective or eating or “substance use”) adj3 (disorder* or condition* or symptom* or assess* or measur*)). ti,ab.
11. ((neuropsy* or behavio?r* or emotion* or mental* or psychiatr* or internalizing or externalizing) adj2 (diagnos* or disorder* or ill or illness*)). ti,ab.
12. or/1‐11
13. exp Child Psychiatry/or exp Child Psychology/
14. exp Adolescent Psychiatry/or exp Adolescent Psychology/
15. (p?ediatric* or child* or adolescen* or youngster* or youth or teen* or boys or girls or neonat* or infan*). ti,ab.
16. pediatrics/
17. 13 or 14 or 15 or 16
18. exp longitudinal studies/or followup studies/or retrospective studies/
19. ((longitudinal or follow‐up or cohort or prospective or retrospective or panel) adj2 stud*). ti,ab.
20. 18 or 19
21. (prevalence or survey* or epidemiolog* or methodol*). ti,ab.
22. epidemiology/or exp morbidity/
23. 21 or 22
24. 12 and 17 and 20 and 23
25. 24 not (animals/not (humans/and animals?.mp.)) [mp = title, abstract, heading word, table of contents, key concepts, original title, tests & measures, mesh]
26. remove duplicates from 24
27. limit 26 to english language
28. limit 27 to (adaptive clinical trial or address or autobiography or bibliography or biography or case reports or clinical study or clinical trial, all or clinical trial, phase i or clinical trial, phase ii or clinical trial, phase iii or clinical trial, phase iv or clinical trial, veterinary or clinical trials, veterinary as topic or clinical trial protocol or clinical trial protocols as topic or clinical trial or comment or congress or consensus development conference or consensus development conference, nih or controlled clinical trial or dataset or dictionary or directory or editorial or equivalence trial or festschrift or government publication or guideline or historical article or interactive tutorial or interview or introductory journal article or lecture or legal case or legislation or letter or news or newspaper article or observational study, veterinary or patient education handout or periodical index or personal narrative or portrait or practice guideline or pragmatic clinical trial or randomized controlled trial or randomized controlled trial, veterinary or technical report or twin study or video‐audio media or webcast) [Limit not valid in APA PsycInfo; records were retained]
29. 27 not 28

### Embase—OVID

1. anxiety disorder/or mental disease/or generalized anxiety disorder/or separation anxiety/
2. attention deficit disorder/
3. depression/or dysthymia/or major depression/
4. conduct disorder/
5. exp eating disorder/
6. oppositional defiant disorder/
7. exp drug dependence/
8. adhd. ti,ab,kw.
9. addiction*.ti,ab,kw.
10. ((mood or anxiety or depress* or attention‐deficit hyperactivity or oppositional‐defiant or conduct or affective or eating or “substance use”) adj3 (disorder* or condition* or symptom* or assess* or measur*)). ti,ab,kw.
11. ((neuropsy* or behavio?r* or emotion* or mental* or psychiatr* or internalizing or externalizing) adj2 (diagnos* or disorder* or ill or illness*)). ti,ab,kw.
12. or/1‐11
13. child/or boy/or girl/or preschool child/or school child/or toddler/
14. adolescent/
15. infant/or baby/or newborn/
16. (p?ediatric* or child* or adolescen* or youngster* or youth or teen* or boys or girls or neonat* or infan*). ti,ab,kw.
17. 13 or 14 or 15 or 16
18. longitudinal study/
19. ((longitudinal or follow‐up or cohort or prospective or retrospective or panel) adj2 stud*). ti,ab,kw.
20. 18 or 19
21. (prevalence or survey* or epidemiolog* or methodol*). ti,ab,kw.
22. epidemiology/or prevalence/
23. 21 or 22
24. 12 and 17 and 20 and 23
25. 24 not (animals/not (humans/and animals?.mp.)) [mp = title, abstract, heading word, drug trade name, original title, device manufacturer, drug manufacturer, device trade name, keyword, floating subheading word, candidate term word]
26. limit 25 to english language
27. limit 26 to (adaptive clinical trial or address or autobiography or bibliography or biography or case reports or clinical study or clinical trial, all or clinical trial, phase i or clinical trial, phase ii or clinical trial, phase iii or clinical trial, phase iv or clinical trial, veterinary or clinical trials, veterinary as topic or clinical trial protocol or clinical trial protocols as topic or clinical trial or comment or congress or consensus development conference or consensus development conference, nih or controlled clinical trial or dataset or dictionary or directory or editorial or equivalence trial or festschrift or government publication or guideline or historical article or interactive tutorial or interview or introductory journal article or lecture or legal case or legislation or letter or news or newspaper article or observational study, veterinary or patient education handout or periodical index or personal narrative or portrait or practice guideline or pragmatic clinical trial or randomized controlled trial or randomized controlled trial, veterinary or technical report or twin study or video‐audio media or webcast) [Limit not valid in APA PsycInfo; records were retained]
28. 26 not 27

## APPENDIX 2 Inclusion/Exclusion criteria

### Inclusion Criteria

Inclusion criteria were as follows: (1) data were from epidemiological longitudinal studies, (2) assessing children aged 0 to 19, (3) for common childhood mental disorders (mood, depressive, anxiety (generalized, separation, social), attention, oppositional defiant, conduct, eating, addiction and substance use) or groupings of emotional, behavioral, internalizing and externalizing. Study information also needed to be available in English or published in an English language peer‐reviewed journal in the form of a methods paper (e.g. protocol, cohort profile, design), prevalence paper (i.e. paper publishing disorder/symptom prevalence in the population of interest), online study website or data user guide to ensure studies included in the analysis could be adequately assessed for eligibility and data extraction.

### Exclusion Criteria

Records that were not subject to peer review, including conference abstracts, presentations, and dissertations or that did not involve primary data collection or data linkage, such as reviews and book chapters, were not included in our review. We also exclude studies that: (1) use adult samples, (2) recruit convenience samples, (3) assess mental disorders using self‐reports of diagnosis or administrative medical records, (4) assessed mental disorders in the context of evaluating a policy or treatment, or clinical practice, or (5) that have a sample size below 50. Further, (6) animal studies, (7) studies that did not collect data at two time points, (8) studies without a parent/caregiver or child informant, and (9) studies where mental disorder symptoms were not measured when at least half of the sample was below the age of 19 were ineligible. Studies focusing on (10) COVID‐19 and other large‐scale disasters, (11) effectiveness of interventions aimed at preventing or treating mental disorders, (12) special populations (clinical or otherwise high‐risk populations, or other special populations or sub‐groups) and (13) the development and testing of new measurement tools were also excluded.

## APPENDIX 3 Data extraction and coding

### Study & Source Information

1. Study ID.
2. Primary information source, coded as methods paper, user guide, website or prevalence paper.
3. Other sources available.
4. Link to source.
5. Language of source.

### Operational Factors

1. Name of study, copied directly from source.
2. Study objectives, copied directly from source.
3. Funding institutions, copied directly from source.
4. Data access, coded as open, restricted, or gated access.
5. Country, coded as name of the country where study was conducted, or “multiple” followed by a list of countries if the study was international.
6. State/Province, coded as name of official jurisdiction directly below the country level or “multiple” if sample selected from more than one jurisdiction.
7. City/Town, coded as name of the city/town where study was conducted or “multiple” if multiple towns/cities included.
8. Geographical unit, coded as international, national, jurisdiction between national and provincial, provincial/state, jurisdiction between national and province/state, jurisdiction below province/state, city/town.
9. Study objective, copied directly from source.
10. Unique features, copied directly from source.

### Temporal Characteristics

1. Year of study initiation.
2. Year of most recent follow‐up.
3. Whether study is ongoing, coded yes/no.
4. Was the study duration at least 12 months, coded yes/no.
5. Number of follow‐ups to date.

### Sampling & Sample Attributes

1. Target population stated, coded yes/no.
2. Target population, copied directly from source.
3. Whether study was a pregnancy cohort, coded yes/no.
4. Baseline sample size.
5. Sample size at last follow‐up.
6. Baseline sample sex, coded as percent of males in the study at baseline.
7. Baseline sample age, coded as mean or range if mean not reported. [Wikipedia was used to convert school grades to age in years when source did not contain sample age]
8. Sample age at last follow‐up, coded as mean or range if mean not reported.
9. Baseline response rate.
10. Sampling approach, coded as stratified, cluster, simple random, total population, or multiple.
11. Sampling frame, copied directly from source.
12. Whether subpopulations were oversampled, coded yes/no.
13. Whether study participants received incentives/gifts for their participation, coded yes/no.

### Mental Disorder Measurement

1. Assessment name used to measure mental disorder symptoms in childhood.
2. Assessment type(s), coded as standardized diagnostic interview, measurement scale, or researcher developed measure.
3. Whether assessment was referenced, coded yes/no.
4. Presence of each of the following mental disorder classes were assessed in childhood: neuro‐developmental/cognitive; psychotic/dissociative; mood/anxiety; obsessive compulsive; trauma/stressor‐related; somatic; eating, elimination, or sleep disorders; sex‐related; gender dysphoria; disruptive, impulse control, or conduct; personality; and substance use/other addictive disorders, coded yes/no.
5. List of the individual disorders assessed, copied directly from source.
6. Informants of assessment: the child, their caregiver, teacher, or service provider, coded yes/no.

### Information Sources, Risk Factors, Protective Factors, and Sequelae

1. Informants of non‐mental health content: the child, their caregiver, teacher, or service provider, coded yes/no.
2. Presence of following data sources: neurocognitive testing, interviewer‐rated content, administrative records, biological‐genetic material, biological (non‐genetic) samples, ecological momentary assessment, observational testing, coded yes/no.
3. Neighbourhood/community factors assessed, coded yes/no.
4. Family‐level risk and protective factors and/or sequelae assessed: family functioning, parenting/partner relationships, socio‐demographic factors, parental adversity, parental mental health/substance use, parental physical health, coded yes/no.
5. Individual‐level risk and protective factors and/or sequelae assessed for the child: birth history/development, physical health, lifestyle, per relationships, school factors, psychological factors, Adverse Childhood Experiences (ACEs), service use, coded yes/no.

## APPENDIX 4 Eligible studies and information sources.

**Table:**

	Study name	Citation
1	(Brazilian) High risk cohort study for psychiatric disorders in childhood	Salum, G. A., Gadelha, A., Pan, P. M., Moriyama, T. S., Graeff‐Martins, A. S., Tamanaha, A. C., Alvarenga, P., Krieger, F. V., Fleitlich‐Bilyk, B., Jackowski, A., Sato, J. R., Brietzke, E., Polanczyk, G. V., Brentani, H., de Jesus Mari, J., Do Rosário, M. C., Manfro, G. G., Bressan, R. A., Mercadante, M. T., … Rohde, L. A. (2015). High risk cohort study for psychiatric disorders in childhood: Rationale, design, methods and preliminary results. International Journal of Methods in Psychiatric Research, 24(1), 58–73. https://doi.org/10.1002/mpr.1459
2	Adolescent Brain Cognitive Development (ABCD) Study	ABCD Study. (2022, August 4). ABCD Study. https://abcdstudy.org/
3	Adolescent Mental Health Cohort study	Litmanen, J., Fröjd, S., Marttunen, M., Isomaa, R., & Kaltiala‐Heino, R. (2017). Are eating disorders and their symptoms increasing in prevalence among adolescent population? Nordic Journal of Psychiatry, 71(1), 61–66. https://doi.org/10.1080/08039488.2016.1224272
4	Alberta Pregnancy Outcomes and Nutrition (APrON) longitudinal study	Letourneau, N., Aghajafari, F., Bell, R. C., Deane, A. J., Dewey, D., Field, C., Giesbrecht, G., Kaplan, B., Leung, B., & Ntanda, H. (2022). The Alberta Pregnancy Outcomes and Nutrition (APrON) longitudinal study: Cohort profile and key findings from the first three years. BMJ Open, 12(2), e047503. https://doi.org/10.1136/bmjopen‐2020‐047503
5	All Our Babies pregnancy cohort	Tough, S. C., McDonald, S. W., Collisson, B. A., Graham, S. A., Kehler, H., Kingston, D., & Benzies, K. (2017). Cohort Profile: The All Our Babies pregnancy cohort (AOB). International Journal of Epidemiology, 46(5), 1389–1390k. https://doi.org/10.1093/ije/dyw363
6	Amsterdam Born Children and their Development (ABCD) Study	van Eijsden, M., Vrijkotte, T. G., Gemke, R. J., & van der Wal, M. F. (2011). Cohort Profile: The Amsterdam Born Children and their Development (ABCD) Study. International Journal of Epidemiology, 40(5), 1176–1186. https://doi.org/10.1093/ije/dyq128
7	Australian Parental Supply of Alcohol Longitudinal Study (APSALS)	Aiken, A., Wadolowski, M., Bruno, R., Najman, J., Kypri, K., Slade, T., Hutchinson, D., McBride, N., & Mattick, R. P. (2017). Cohort Profile: The Australian Parental Supply of Alcohol Longitudinal Study (APSALS). International Journal of Epidemiology, 46(2), e6. https://doi.org/10.1093/ije/dyv051
8	Australian Temperament Project	Home—The Australian Temperament Project. (2022, August 5). https://www.melbournechildrens.com/atp/
9	Avon Longitudinal Study of Parents and Children (ALSPAC)	Golding, Pembrey, Jones, & Team, T. A. S. (2001). ALSPAC–The Avon Longitudinal Study of Parents and Children. Paediatric and Perinatal Epidemiology, 15(1), 74–87. https://doi.org/10.1046/j.1365‐3016.2001.00325.x
10	Babies after SCOPE/Cork BASELINE Birth Cohort Study	O’Donovan, S. M., Murray, D. M., Hourihane, J. O., Kenny, L. C., Irvine, A. D., & Kiely, M. (2015). Cohort profile: The Cork BASELINE Birth Cohort Study: Babies after SCOPE: Evaluating the Longitudinal Impact on Neurological and Nutritional Endpoints. International Journal of Epidemiology, 44(3), 764–775. https://doi.org/10.1093/ije/dyu157
11	Barwon Infant Study	Vuillermin, P., Saffery, R., Allen, K. J., Carlin, J. B., Tang, M. L., Ranganathan, S., Burgner, D., Dwyer, T., Collier, F., Jachno, K., Sly, P., Symeonides, C., McCloskey, K., Molloy, J., Forrester, M., & Ponsonby, A.‐L. (2015). Cohort Profile: The Barwon Infant Study. International Journal of Epidemiology, 44(4), 1148–1160. https://doi.org/10.1093/ije/dyv026
12	Behavior and Mind Health (BeMIND) study	Beesdo‐Baum, K., Voss, C., Venz, J., Hoyer, J., Berwanger, J., Kische, H., Ollmann, T. M., & Pieper, L. (2020). The Behavior and Mind Health (BeMIND) study: Methods, design and baseline sample characteristics of a cohort study among adolescents and young adults. International Journal of Methods in Psychiatric Research, 29(1), e1804. https://doi.org/10.1002/mpr.1804
13	Belfast Youth Development Study (BYDS)	Higgins, K., McLaughlin, A., Perra, O., McCartan, C., McCann, M., Percy, A., & Jordan, J.‐A. (2018). The Belfast Youth Development Study (BYDS): A prospective cohort study of the initiation, persistence and desistance of substance use from adolescence to adulthood in Northern Ireland. PLOS ONE, 13(5), e0195192. https://doi.org/10.1371/journal.pone.0195192
14	BELLA study	Otto, C., Reiss, F., Voss, C., Wüstner, A., Meyrose, A.‐K., Hölling, H., & Ravens‐Sieberer, U. (2021). Mental health and well‐being from childhood to adulthood: Design, methods and results of the 11‐year follow‐up of the BELLA study. European Child & Adolescent Psychiatry, 30(10), 1559–1577. https://doi.org/10.1007/s00787‐020‐01630‐4
15	Bergen Child Study	The Bergen Child Study—Norce. (2022, August 5). NORCE Norwegian Research Centre. https://www.norceresearch.no/en/projects/the‐bergen‐child‐study
16	Birth to Twenty	Richter, L., Norris, S., Pettifor, J., Yach, D., & Cameron, N. (2007). Cohort Profile: Mandela's children: The 1990 birth to twenty study in South Africa. International Journal of Epidemiology, 36(3), 504–511. https://doi.org/10.1093/ije/dym016
17	Born in Bradford (BiB)	Wright, J., Small, N., Raynor, P., Tuffnell, D., Bhopal, R., Cameron, N., Fairley, L., Lawlor, D. A., Parslow, R., Petherick, E. S., Pickett, K. E., Waiblinger, D., West, J., & on behalf of the Born in Bradford Scientific Collaborators Group. (2013). Cohort Profile: The Born in Bradford multi‐ethnic family cohort study. International Journal of Epidemiology, 42(4), 978–991. https://doi.org/10.1093/ije/dys112
18	British Cohort Study	BCS70. (2022, August 5). https://bcs70.info/
19	British Household Panel Survey (BHPS)	British Household Panel Survey (BHPS)—Institute for Social and Economic Research (ISER). (2022, August 5). https://www.iser.essex.ac.uk/bhps/
20	Canadian Healthy Infant Longitudinal Development (CHILD) Cohort Study	About—CHILD Cohort Study. (2022, August 5). https://childstudy.ca/about/
21	Challenging Times study	Harley, M. E., Connor, D., Clarke, M. C., Kelleher, I., Coughlan, H., Lynch, F., Fitzpatrick, C., & Cannon, M. (2015). Prevalence of Mental Disorder among young adults in Ireland: A population based study. Irish Journal of Psychological Medicine, 32(1), 79–91. https://doi.org/10.1017/ipm.2014.88
22	Childhood to Adolescence Transition Study (CATS)	Mundy, L. K., Simmons, J. G., Allen, N. B., Viner, R. M., Bayer, J. K., Olds, T., Williams, J., Olsson, C., Romaniuk, H., Mensah, F., Sawyer, S. M., Degenhardt, L., Alati, R., Wake, M., Jacka, F., & Patton, G. C. (2013). Study protocol: The Childhood to Adolescence Transition Study (CATS). BMC Pediatrics, 13(1), 160. https://doi.org/10.1186/1471‐2431‐13‐160
23	Children in the Community Study of Developmental Course of Personality Disorder	Cohen, P., Crawford, T. N., Johnson, J. G., & Kasen, S. (2005). The Children in the Community Study of Developmental Course of Personality Disorder. Journal of Personality Disorders, 19(5), 466–486. https://doi.org/10.1521/pedi.2005.19.5.466
24	Children of 1997	Schooling, C. M., Hui, L. L., Ho, L. M., Lam, T.‐H., & Leung, G. M. (2012). Cohort Profile: ‘Children of 1997’: a Hong Kong Chinese birth cohort. International Journal of Epidemiology, 41(3), 611–620. https://doi.org/10.1093/ije/dyq243
25	China Education Panel Survey	Ma, L., Gao, L., Chiu, D. T., Ding, Y., Wang, W., & Wang, Y. (2020). Depressive symptoms prevalence, associated family factors, and gender differences: A national cohort study of middle school students in China. Journal of Affective Disorders, 274, 545–552. https://doi.org/10.1016/j.jad.2020.05.128
26	China‐Anhui birth cohort study	Cohort Profile: The China‐Anhui birth cohort study | International Journal of Epidemiology | Oxford Academic. (2022, August 7). https://academic.oup.com/ije/article/42/3/709/910067
27	Christchurch Health and Development Study	Fergusson, D. M., & Horwood, J. L. (2001). The Christchurch Health and Development Study: Review of Findings on Child and Adolescent Mental Health. Australian & New Zealand Journal of Psychiatry, 35(3), 287–296. https://doi.org/10.1046/j.1440‐1614.2001.00902.x
28	Conditions Affecting Neurocognitive Development and Learning in Early Childhood (CANDLE) Study	CANDLE Study. (2022, August 7). CANDLE Study. https://candlestudy.uthsc.edu/
29	Connecticut Early Development Project	Carter, A. S., Wagmiller, R. J., Gray, S. A. O., McCarthy, K. J., Horwitz, S. M., & Briggs‐Gowan, M. J. (2010). Prevalence of DSM‐IV disorder in a representative, healthy birth cohort at school entry: Sociodemographic risks and social adaptation. Journal of the American Academy of Child & Adolescent Psychiatry, 49(7), 686–698. https://doi.org/10.1097/00004583‐201007000‐00009
30	Copenhagen Child Cohort Study (CCC2000)	Olsen, E. M., Rask, C. U., Elberling, H., Jeppesen, P., Clemmensen, L., Munkholm, A., Li, X. Q., Hansen, M. H., Rimvall, M. K., Linneberg, A., Munch, I. C., Larsen, M., Jørgensen, T., & Skovgaard, A. M. (2020). Cohort Profile: The Copenhagen Child Cohort Study (CCC2000). International Journal of Epidemiology, 49(2), 370–371l. https://doi.org/10.1093/ije/dyz256
31	Crakow Research Project on Adolescent Depression	Modrzejewska, R., Bomba, J., & Pac, A. (2019). Depressive symptoms among adolescents in non clinical Krakow's population—thirty years' follow‐up. Psychiatria Polska, 53(4), 723–735. https://doi.org/10.12740/PP/99536
32	Danish Longitudinal Survey of Children (DALSC)	Ottosen, M. H. (2011). Research on the Danish Longitudinal Survey of Children (DALSC) at the Danish National Centre for Social Research. Scandinavian Journal of Public Health, 39(7_suppl), 121–125. https://doi.org/10.1177/1403494811399165
33	Drakenstein Child Health Study (DCHS)	Donald, K. A., Hoogenhout, M., Plooy, C. P. du, Wedderburn, C. J., Nhapi, R. T., Barnett, W., Hoffman, N., Malcolm‐Smith, S., Zar, H. J., & Stein, D. J. (2018). Drakenstein Child Health Study (DCHS): Investigating determinants of early child development and cognition. BMJ Paediatrics Open, 2(1), e000282. https://doi.org/10.1136/bmjpo‐2018‐000282
34	Dunedin Multidisciplinary Health and Development Study	Poulton, R., Moffitt, T. E., & Silva, P. A. (2015). The Dunedin Multidisciplinary Health and Development Study: Overview of the first 40 years, with an eye to the future. Social Psychiatry and Psychiatric Epidemiology, 50(5), 679–693. https://doi.org/10.1007/s00127‐015‐1048‐8
35	Dutch ‘TRacking Adolescents’ Individual Lives' Survey’ (TRAILS)	Huisman, M., Oldehinkel, A. J., de Winter, A., Minderaa, R. B., de Bildt, A., Huizink, A. C., Verhulst, F. C., & Ormel, J. (2008). Cohort Profile: The Dutch ‘TRacking Adolescents’ Individual Lives' Survey’; TRAILS. International Journal of Epidemiology, 37(6), 1227–1235. https://doi.org/10.1093/ije/dym273
36	Early Child Care and Youth Development	Study of Early Child Care and Youth Development (SECCYD) Overview (Historical/For Reference Only). (2022, August 5). https://www.nichd.nih.gov/research/supported/seccyd/overview
37	Early Childhood Longitudinal Studies Program (ECLS)—Kindergarten Class of 1998‐99 (ECLS‐K)	Early Childhood Longitudinal Studies Program (ECLS)—Kindergarten Class of 1998‐99 (ECLS‐K). (2022, August 7). National Center for Education Statistics. https://nces.ed.gov/ecls/kindergarten.asp
38	Early Childhood Longitudinal Studies Program (ECLS)—Kindergarten Class of 2010‐11 (ECLS‐K:2011)	Early Childhood Longitudinal Studies Program (ECLS)—Kindergarten Class of 2010‐11 (ECLS‐K:2011). (2022, August 7). National Center for Education Statistics. https://nces.ed.gov/ecls/kindergarten2011.asp
39	Early Childhood Longitudinal Study, Birth Cohort (ECLS‐B)	Andreassen, C., & Fletcher, P. (2007). Early Childhood Longitudinal Study, Birth Cohort (ECLS‐B): Psychometric Report for the 2‐Year Data Collection. Methodology Report. NCES 2007‐084. In National Center for Education Statistics. National Center for Education Statistics. https://eric.ed.gov/?id=ED497762
40	Early Developmental Stages of Psychopathology Study (EDSP)	Wittchen, H.‐U., Perkonigg, A., Lachner, G., & Nelson, C. B. (1998). Early Developmental Stages of Psychopathology Study (EDSP): Objectives and Design. European Addiction Research, 4(1–2), 18–27. https://doi.org/10.1159/000018921
41	EAT 2010–2018	About—Project EAT ‐ School of Public Health—University of Minnesota. (2022b, August 7). School of Public Health. https://www.sph.umn.edu/research/projects/project‐eat/about/
42	EDEN mother‐child cohort study	Heude, B., Forhan, A., Slama, R., Douhaud, L., Bedel, S., Saurel‐Cubizolles, M.‐J., Hankard, R., Thiebaugeorges, O., De Agostini, M., Annesi‐Maesano, I., Kaminski, M., Charles, M.‐A., Annesi‐Maesano, I., Bernard, J., Botton, J., Charles, M.‐A., Dargent‐Molina, P., de Lauzon‐Guillain, B., Ducimetière, P., … on behalf of the EDEN mother‐child cohort study group. (2016). Cohort Profile: The EDEN mother‐child cohort on the prenatal and early postnatal determinants of child health and development. International Journal of Epidemiology, 45(2), 353–363. https://doi.org/10.1093/ije/dyv151
43	Environment and Development of Children (EDC) study	Kim, K.‐N., Lim, Y.‐H., Shin, C. H., Lee, Y. A., Kim, B.‐N., Kim, J. I., Hwang, I. G., Hwang, M. S., Suh, J.‐H., & Hong, Y.‐C. (2018). Cohort Profile: The Environment and Development of Children (EDC) study: a prospective children's cohort. International Journal of Epidemiology, 47(4), 1049–1050f. https://doi.org/10.1093/ije/dyy070
44	Epidemiological Multicenter Child Psychiatric Study	Almqvist, F., Ikäheimo, K., Kumpulainen, K., Tuompo‐Johansson, E., Linna, S. L., Puura, K., Moilanen, I., Räsänen, E., Tamminen, T., & Piha, J. (1999a). Design and subjects of a Finnish epidemiological study on psychiatric disorders in childhood. European Child & Adolescent Psychiatry, 8 Suppl 4, 3–6. https://doi.org/10.1007/pl00010697
45	EPITeen	User, S. (2022, August 7). Página principal. http://epiteen.iscsp.ulisboa.pt/
46	Estonian Children Personality, Behaviour and Health Study (ECPBHS)	Original ECPBHS. (2022, August 7). ELIKTU. http://www.ecpbhs.ee/en/data‐collection/original‐ecpbhs/
47	European Longitudinal Study of Pregnancy and Childhood (ELSPAC)	Piler, P., Kandrnal, V., Kukla, L., Andrýsková, L., Švancara, J., Jarkovský, J., Dušek, L., Pikhart, H., Bobák, M., & Klánová, J. (2017). Cohort Profile: The European Longitudinal Study of Pregnancy and Childhood (ELSPAC) in the Czech Republic. International Journal of Epidemiology, 46(5), 1379–1379f. https://doi.org/10.1093/ije/dyw091
48	Evaluation of infant and young child psychotherapy through prevalence and intervention studies (SKKIPPI)	Fricke, J., Bolster, M., Ludwig‐Körner, C., Kuchinke, L., Schlensog‐Schuster, F., Vienhues, P., Reinhold, T., Berghöfer, A., Roll, S., & Keil, T. (2021). Occurrence and determinants of parental psychosocial stress and mental health disorders in parents and their children in early childhood: Rationale, objectives, and design of the population‐based SKKIPPI cohort study. Social Psychiatry and Psychiatric Epidemiology, 56(6), 1103–1112. https://doi.org/10.1007/s00127‐020‐02004‐6
49	EveryBODY Study	Burt, A., Mitchison, D., Dale, E., Bussey, K., Trompeter, N., Lonergan, A., & Hay, P. (2020). Prevalence, features and health impacts of eating disorders amongst First‐Australian Yiramarang (adolescents) and in comparison with other Australian adolescents. Journal of Eating Disorders, 8(1), 10. https://doi.org/10.1186/s40337‐020‐0286‐7
50	Ewha Birth and Growth Study	Lee, H. A., Park, B., Min, J., Choi, E. J., Kim, U. J., Park, H. J., Park, E. A., Cho, S. J., Kim, H. S., Lee, H., Kim, Y. J., Hong, Y. S., Kim, E.‐J., Ha, E. H., & Park, H. (2021). Cohort profile: The Ewha Birth and Growth Study. Epidemiology and Health, 43, e2021016. https://doi.org/10.4178/epih.e2021016
51	Family Life Project	Vernon‐Feagans, L., Cox, M., Willoughby, M., Burchinal, M., Garrett‐Peters, P., Mills‐Koonce, R., Garrett‐Peiers, P., Conger, R. D., & Bauer, P. J. (2013). THE FAMILY LIFE PROJECT: AN EPIDEMIOLOGICAL AND DEVELOPMENTAL STUDY OF YOUNG CHILDREN LIVING IN POOR RURAL COMMUNITIES. Monographs of the Society for Research in Child Development, 78(5), i–150.
52	Finnish 1981 Birth Cohort	Almqvist, F., Ikäheimo, K., Kumpulainen, K., Tuompo‐Johansson, E., Linna, S. L., Puura, K., Moilanen, I., Räsänen, E., Tamminen, T., & Piha, J. (1999b). Design and subjects of a Finnish epidemiological study on psychiatric disorders in childhood. European Child & Adolescent Psychiatry, 8(4), S3. https://doi.org/10.1007/PL00010697
53	Finnish Family Competence (FFC) cohort study	Pihlakoski, L., Sourander, A., Aromaa, M., Rönning, J. A., Rautava, P., Helenius, H., & Sillanpää, M. (2013). Do Antenatal and Postnatal Parental Psychological Distress, and Recognized Need of Help Predict Preadolescent's Psychiatric Symptoms? The Finnish Family Competence Cohort Study. Child Psychiatry & Human Development, 44(2), 305–319. https://doi.org/10.1007/s10578‐012‐0326‐x
54	Finnish Health in Teens (Fin‐HIT) study	Figueiredo, R. A. de O., Simola‐Ström, S., Rounge, T. B., Viljakainen, H., Eriksson, J. G., Roos, E., & Weiderpass, E. (2019). Cohort Profile: The Finnish Health in Teens (Fin‐HIT) study: a population‐based study. International Journal of Epidemiology, 48(1), 23–24h. https://doi.org/10.1093/ije/dyy189
55	Fragile Families and Child Wellbeing	Reichman, N. E., Teitler, J. O., Garfinkel, I., & McLanahan, S. S. (2001). Fragile Families: Sample and design. Children and Youth Services Review, 23(4–5), 303–326. https://doi.org/10.1016/S0190‐7409(01)00141‐4
56	Gateshead Millennium Study	Parkinson, K. N., Pearce, M. S., Dale, A., Reilly, J. J., Drewett, R. F., Wright, C. M., Relton, C. L., McArdle, P., Le Couteur, A. S., & Adamson, A. J. (2011). Cohort Profile: The Gateshead Millennium Study. International Journal of Epidemiology, 40(2), 308–317. https://doi.org/10.1093/ije/dyq015
57	Generation R Study	Kooijman, M. N., Kruithof, C. J., van Duijn, C. M., Duijts, L., Franco, O. H., van IJzendoorn, M. H., de Jongste, J. C., Klaver, C. C. W., van der Lugt, A., Mackenbach, J. P., Moll, H. A., Peeters, R. P., Raat, H., Rings, E. H. H. M., Rivadeneira, F., van der Schroeff, M. P., Steegers, E. A. P., Tiemeier, H., Uitterlinden, A. G., … Jaddoe, V. W. V. (2016). The Generation R Study: Design and cohort update 2017. European Journal of Epidemiology, 31(12), 1243–1264. https://doi.org/10.1007/s10654‐016‐0224‐9
58	German Health Interview and Examination Survey for Children and Adolescents (KiGGS)	Mauz, E., Lange, M., Houben, R., Hoffmann, R., Allen, J., Gößwald, A., Hölling, H., Lampert, T., Lange, C., Poethko‐Müller, C., Richter, A., Rosario, A. S., von Schenck, U., Ziese, T., Kurth, B.‐M., & Team, on behalf of the K. C. R. (2020). Cohort profile: KiGGS cohort longitudinal study on the health of children, adolescents and young adults in Germany. International Journal of Epidemiology, 49(2), 375–375k. https://doi.org/10.1093/ije/dyz231
59	Great Smoky Mountains Study of Youth	Costello, E. J., Angold, A., Burns, B. J., Stangl, D. K., Tweed, D. L., Erkanli, A., & Worthman, C. M. (1996). The Great Smoky Mountains Study of Youth: Goals, Design, Methods, and the Prevalence of DSM‐III‐R Disorders. Archives of General Psychiatry, 53(12), 1129–1136. https://doi.org/10.1001/archpsyc.1996.01830120067012
60	Growing Up in Ireland/National Longitudinal Study of Children	Growing Up in Ireland—National Longitudinal Study of Children. (2022, August 7). https://www.growingup.ie/
61	Growing Up in New Zealand	Morton, S. M. B., Atatoa Carr, P. E., Grant, C. C., Robinson, E. M., Bandara, D. K., Bird, A., Ivory, V. C., Kingi, T. K. R., Liang, R., Marks, E. J., Perese, L. M., Peterson, E. R., Pryor, J. E., Reese, E., Schmidt, J. M., Waldie, K. E., & Wall, C. (2013). Cohort Profile: Growing Up in New Zealand. International Journal of Epidemiology, 42(1), 65–75. https://doi.org/10.1093/ije/dyr206
62	Growing Up in Scotland	Growing Up in Scotland | following the lives of Scotland's children. (2022, August 7). https://growingupinscotland.org.uk/
63	Growing Up Today Study II	About‐guts. (2022, August 7). https://gutsweb.org/about‐guts/
64	Hamamatsu Birth Cohort for Mothers and Children (HBC Study)	Takagai, S., Tsuchiya, K. J., Itoh, H., Kanayama, N., Mori, N., Takei, N., & on behalf of HBC Study Team. (2016). Cohort Profile: Hamamatsu Birth Cohort for Mothers and Children (HBC Study). International Journal of Epidemiology, 45(2), 333–342. https://doi.org/10.1093/ije/dyv290
65	Happiness & Health Study	Vogel, E. A., Cho, J., McConnell, R. S., Barrington‐Trimis, J. L., & Leventhal, A. M. (2020). Prevalence of Electronic Cigarette Dependence Among Youth and Its Association With Future Use. JAMA Network Open, 3(2), e1921513. https://doi.org/10.1001/jamanetworkopen.2019.21513
66	Healthy Passages	Windle, M., Grunbaum, J. A., Elliott, M., Tortolero, S. R., Berry, S., Gilliland, J., Kanouse, D. E., Parcel, G. S., Wallander, J., Kelder, S., Collins, J., Kolbe, L., & Schuster, M. (2004). Healthy passages. A multilevel, multimethod longitudinal study of adolescent health. American Journal of Preventive Medicine, 27(2), 164–172. https://doi.org/10.1016/j.amepre.2004.04.007
67	Helsinki Birth Cohort Study	Helsinki Birth Cohort Study. (2022, August 7). JPND. https://www.neurodegenerationresearch.eu/cohort/helsinki‐birth‐cohort‐study/
68	Hokkaido Study on Environment and Children's Health‐Hokkaido cohort	Kishi, R., Ikeda‐Araki, A., Miyashita, C., Itoh, S., Kobayashi, S., Ait Bamai, Y., Yamazaki, K., Tamura, N., Minatoya, M., Ketema, R. M., Poudel, K., Miura, R., Masuda, H., Itoh, M., Yamaguchi, T., Fukunaga, H., Ito, K., Goudarzi, H., Sasaki, S., … the members of The Hokkaido Study on Environment and Children's Health. (2021). Hokkaido birth cohort study on environment and children's health: Cohort profile 2021. Environmental Health and Preventive Medicine, 26(1), 59. https://doi.org/10.1186/s12199‐021‐00980‐y
69	Hokkaido Study on Environment and Children's Health‐Sapparo cohort	Kishi, R., Ikeda‐Araki, A., Miyashita, C., Itoh, S., Kobayashi, S., Ait Bamai, Y., Yamazaki, K., Tamura, N., Minatoya, M., Ketema, R. M., Poudel, K., Miura, R., Masuda, H., Itoh, M., Yamaguchi, T., Fukunaga, H., Ito, K., Goudarzi, H., Sasaki, S., … the members of The Hokkaido Study on Environment and Children's Health. (2021). Hokkaido birth cohort study on environment and children's health: Cohort profile 2021. Environmental Health and Preventive Medicine, 26(1), 59. https://doi.org/10.1186/s12199‐021‐00980‐y
70	IMAGEN study	Mascarell Maričić, L., Walter, H., Rosenthal, A., Ripke, S., Quinlan, E. B., Banaschewski, T., Barker, G. J., Bokde, A. L. W., Bromberg, U., Büchel, C., Desrivières, S., Flor, H., Frouin, V., Garavan, H., Itterman, B., Martinot, J.‐L., Martinot, M.‐L. P., Nees, F., Orfanos, D. P., … Heinz, A. (2020). The IMAGEN study: A decade of imaging genetics in adolescents. Molecular Psychiatry, 25(11), 2648–2671. https://doi.org/10.1038/s41380‐020‐0822‐5
71	INfancia y Medio Ambiente (INMA)/Environment and Childhood project	Gascon, M., Guxens, M., Vrijheid, M., Torrent, M., Ibarluzea, J., Fano, E., Llop, S., Ballester, F., Fernández, M. F., Tardón, A., Fernández‐Somoano, A., & Sunyer, J. (2017). The INMA—INfancia y Medio Ambiente—(Environment and Childhood) project: More than 10 years contributing to environmental and neuropsychological research. International Journal of Hygiene and Environmental Health, 220(4), 647–658. https://doi.org/10.1016/j.ijheh.2017.02.008
72	International Youth Development Study (IYDS)	International Youth Development Study (IYDS)—LifeCourse. (2022, August 7). https://lifecourse.melbournechildrens.com/cohorts/iyds/#overview
73	Japan environment and children's study (JECS)	Kawamoto, T., Nitta, H., Murata, K., Toda, E., Tsukamoto, N., Hasegawa, M., Yamagata, Z., Kayama, F., Kishi, R., Ohya, Y., Saito, H., Sago, H., Okuyama, M., Ogata, T., Yokoya, S., Koresawa, Y., Shibata, Y., Nakayama, S., Michikawa, T., … Satoh, H. (2014). Rationale and study design of the Japan environment and children's study (JECS). BMC Public Health, 14(1), 1–8. https://doi.org/10.1186/1471‐2458‐14‐25
74	Jimma Longitudinal Family Survey of Youth (JLFSY)	JLFSY Study Design | Jimma Longitudinal Family Survey of Youth (JLFSY). (2022, August 7). https://www.brown.edu/research/projects/jimma‐longitudinal‐family‐survey‐of‐youth/jlfsy‐study‐design
75	Knowledge of adolescents' mental health and learning (KUPOL)	Research project KUPOL—Knowledge of adolescents' mental health and learning | EPHIR | Karolinska Institutet. (2022, August 7). https://ki.se/en/gph/research‐project‐kupol‐knowledge‐of‐adolescents‐mental‐health‐and‐learning‐ephir
76	Korea Welfare Panel Study (KOWEPS)	KOWEPS ‐ Korea Welfare Panel Study. (2022, August 7). https://www.koweps.re.kr:442/eng/about/area.do
77	Korea Youth Panel Survey (KYPS)	National Youth Policy Institute. (2022). Korea Youth Panel Survey (KYPS) User's Guide For 1st‐5th Year of Panel Study of 4th grade Elementary School Youths. https://view.officeapps.live.com/op/view.aspx?src=https%3A%2F%2Fkossda.snu.ac.kr%2Fbitstream%2F20.500.12236%2F13414%2F7%2Feng_user%2527s%2520guide_2004_2008.doc&wdOrigin=BROWSELINK
78	Korean Children and Youth Panel Survey	NYPI Youth and Children Data Archive. (2022, August 7). https://www.nypi.re.kr/archive/board?menuId=MENU00329
79	LIFECOURSE study	Halldorsdottir, T., Kristjansson, A. L., Asgeirsdottir, B. B., Thorisdottir, I. E., Sigfusson, J., Tolgyes, E. M. J., Valdimarsdottir, H. B., Allegrante, J., & Sigfusdottir, I. D. (2021). A multi‐level developmental approach towards understanding adolescent mental health and behaviour: Rationale, design and methods of the LIFECOURSE study in Iceland. Social Psychiatry and Psychiatric Epidemiology, 56(3), 519–529. https://doi.org/10.1007/s00127‐020‐01995‐6
80	LISAplus	Heinrich, J., Brüske, I., Cramer, C., Hoffmann, U., Schnappinger, M., Schaaf, B., von Berg, A., Berdel, D., Krämer, U., Lehmann, I., Herbarth, O., Borte, M., Grübl, A., Bauer, C. P., Beckmann, C., Behrendt, H., Ring, J., & Koletzko, S. (2017). GINIplus and LISAplus—Design and selected results of two German birth cohorts about natural course of atopic diseases and their determinants. Allergologie Select, 1(1), 85–95. https://doi.org/10.5414/ALX01455E
81	Longitudinal Research on Development In Adolescence (LoRDIA)	Ahlgren, T., Kalin, T., & Gerdner, A. (2021). Self‐rated child maltreatment, behavioural problems, and contacts with welfare and police authorities—longitudinal community data. European Journal of Social Work, 24(4), 642–656. https://doi.org/10.1080/13691457.2021.1896996
82	Longitudinal Study of Australian Children/Growing up in Australia	Longitudinal Study of Australian Children. (2022, August 5). https://growingupinaustralia.gov.au/
83	Mater‐University of Queensland Study of Pregnancy (MUSP)	Najman, J. M., Alati, R., Bor, W., Clavarino, A., Mamun, A., McGrath, J. J., McIntyre, D., O’Callaghan, M., Scott, J., Shuttlewood, G., Williams, G. M., & Wray, N. (2015). Cohort Profile Update: The Mater‐University of Queensland Study of Pregnancy (MUSP). International Journal of Epidemiology, 44(1), 78–78f. https://doi.org/10.1093/ije/dyu234
84	Maternal Adversity, Vulnerability and Neurodevelopment Project	O’Donnell, K. A., Gaudreau, H., Colalillo, S., Steiner, M., Atkinson, L., Moss, E., Goldberg, S., Karama, S., Matthews, S. G., Lydon, J. E., Silveira, P. P., Wazana, A. D., Levitan, R. D., Sokolowski, M. B., Kennedy, J. L., Fleming, A., & Meaney, M. J. (2014). The Maternal Adversity, Vulnerability and Neurodevelopment Project: Theory and Methodology. Canadian Journal of Psychiatry. Revue Canadienne de Psychiatrie, 59(9), 497–508.
85	Medical Research Council National Survey of Health and Development/MRC National Survey of Health and Development/NSHD/British 1946 Birth Cohort/British Cohort 1946	Wadsworth, M., Kuh, D., Richards, M., & Hardy, R. (2006). Cohort Profile: The 1946 National Birth Cohort (MRC National Survey of Health and Development). International Journal of Epidemiology, 35(1), 49–54. https://doi.org/10.1093/ije/dyi201
86	Millennium Cohort Study (MCS)	Connelly, R., & Platt, L. (2014). Cohort Profile: UK Millennium Cohort Study (MCS). International Journal of Epidemiology, 43(6), 1719–1725. https://doi.org/10.1093/ije/dyu001
87	Monitoring young lifestyles (MyLife)	Brunborg, G. S., Scheffels, J., Tokle, R., Buvik, K., Kvaavik, E., & Burdzovic Andreas, J. (2019). Monitoring young lifestyles (MyLife)—A prospective longitudinal quantitative and qualitative study of youth development and substance use in Norway. BMJ Open, 9(10), e031084. https://doi.org/10.1136/bmjopen‐2019‐031084
88	Multiple Opportunities to Reach Excellence (MORE) Project	Cooley‐Strickland, M., Quille, T. J., Griffin, R. S., Stuart, E. A., Bradshaw, C. P., & Furr‐Holden, D. (2009). Community Violence and Youth: Affect, Behavior, Substance Use, and Academics. Clinical Child and Family Psychology Review, 12(2), 127–156. https://doi.org/10.1007/s10567‐009‐0051‐6
89	National Child Development Study	1958 National Child Development Study. (2022, August 7). CLOSER. https://www.closer.ac.uk/study/1958‐national‐child‐development‐study/
90	National Collaborative Perinatal Project (NCPP)	Hardy, J. B. (1971). The Johns Hopkins Collaborative Perinatal Project. Descriptive background. The Johns Hopkins Medical Journal, 128(5), 238–243.
91	National Longitudinal Study of Adolescent to Adult Health (Add Health)	Harris, K. M., Halpern, C. T., Whitsel, E. A., Hussey, J. M., Killeya‐Jones, L. A., Tabor, J., & Dean, S. C. (2019). Cohort Profile: The National Longitudinal Study of Adolescent to Adult Health (Add Health). International Journal of Epidemiology, 48(5), 1415–1415k. https://doi.org/10.1093/ije/dyz115
92	National Longitudinal Survey of Children and Youth (NLSCY)	Government of Canada, S. C. (2009, May 14). National Longitudinal Survey of Children and Youth (NLSCY). https://www23.statcan.gc.ca/imdb/p2SV.pl?Function=getSurvey&SDDS=4450
93	National Longitudinal Survey of Youth 1979 (NLSY79)	Rothstein, D. S., Carr, D., & Cooksey, E. (2019). Cohort Profile: The National Longitudinal Survey of Youth 1979 (NLSY79). International Journal of Epidemiology, 48(1), 22–22e. https://doi.org/10.1093/ije/dyy133
94	National Survey of Families and Households (NSFH)	National Survey of Families and Households (NSFH). (2022, August 7). https://www.ssc.wisc.edu/nsfh/content1.htm
95	NEXT Generation Health Study	Goldstein, R. B., Lee, A. K., Haynie, D. L., Luk, J. W., Fairman, B. J., Liu, D., Jeffers, J. S., Simons‐Morton, B. G., & Gilman, S. E. (2019). Neighbourhood disadvantage and depressive symptoms among adolescents followed into emerging adulthood. J Epidemiol Community Health, 73(7), 590–597. https://doi.org/10.1136/jech‐2018‐212004
96	Next Steps/Longitudinal Study of Young People in England (LSYPE)	CLS | Next Steps. (2022, August 7). https://cls.ucl.ac.uk/cls‐studies/next‐steps/
97	Nicotine Dependence in Teens (NDIT) Study	O’Loughlin, J., Dugas, E. N., Brunet, J., DiFranza, J., Engert, J. C., Gervais, A., Gray‐Donald, K., Karp, I., Low, N. C., Sabiston, C., Sylvestre, M.‐P., Tyndale, R. F., Auger, N., Auger, N., Mathieu, B., Tracie, B., Chaiton, M., Chenoweth, M. J., Constantin, E., … Paradis, G. (2015). Cohort Profile: The Nicotine Dependence in Teens (NDIT) Study. International Journal of Epidemiology, 44(5), 1537–1546. https://doi.org/10.1093/ije/dyu135
98	No Study Name	Chi, X., Liu, X., Huang, Q., Huang, L., Zhang, P., & Chen, X. (2020). Depressive Symptoms among Junior High School Students in Southern China: Prevalence, Changes, and Psychosocial Correlates. Journal of Affective Disorders, 274, 1191–1200. https://doi.org/10.1016/j.jad.2020.05.034
99	No Study Name	de la Osa, N., Penelo, E., Navarro, J. B., Trepat, E., & Ezpeleta, L. (2019). Prevalence, comorbidity, functioning and long‐term effects of subthreshold oppositional defiant disorder in a community sample of preschoolers. European Child & Adolescent Psychiatry, 28(10), 1385–1393. https://doi.org/10.1007/s00787‐019‐01300‐0
100	No Study Name	Fu, K.‐Wa., Chan, W. S. C., Wong, P. W. C., & Yip, P. S. F. (2010). Internet addiction: Prevalence, discriminant validity and correlates among adolescents in Hong Kong. British Journal of Psychiatry, 196(6), 486–492. https://doi.org/10.1192/bjp.bp.109.075002
101	No Study Name	Furniss, T., Beyer, T., & Guggenmos, J. (2006). Prevalence of behavioural and emotional problems among six‐years‐old preschool children. Social Psychiatry and Psychiatric Epidemiology, 41(5), 394–399. https://doi.org/10.1007/s00127‐006‐0045‐3
102	No Study Name	Garisch, J. A., & Wilson, M. S. (2015). Prevalence, correlates, and prospective predictors of non‐suicidal self‐injury among New Zealand adolescents: Cross‐sectional and longitudinal survey data. Child and Adolescent Psychiatry and Mental Health, 9(1), 28. https://doi.org/10.1186/s13034‐015‐0055‐6
103	No Study Name	Isomaa, R., Isomaa, A.‐L., Marttunen, M., Kaltiala‐Heino, R., & Björkqvist, K. (2009). The prevalence, incidence and development of eating disorders in Finnish adolescents: A two‐step 3‐year follow‐up study. European Eating Disorders Review: The Journal of the Eating Disorders Association, 17(3), 199–207. https://doi.org/10.1002/erv.919
104	No Study Name	Larson, C. P., Pless, I. B., & Miettinen, O. (1988). Preschool behavior disorders: Their prevalence in relation to determinants. The Journal of Pediatrics, 113(2), 278–285. https://doi.org/10.1016/s0022‐3476(88)80265‐8
105	No Study Name	Rojo‐Moreno, L., Arribas, P., Plumed, J., Gimeno, N., García‐Blanco, A., Vaz‐Leal, F., Luisa Vila, M., & Livianos, L. (2015). Prevalence and comorbidity of eating disorders among a community sample of adolescents: 2‐year follow‐up. Psychiatry Research, 227(1), 52–57. https://doi.org/10.1016/j.psychres.2015.02.015
106	No Study Name	Ezpeleta, L., Guillamón, N., Granero, R., de la Osa, N., María Domènech, J., & Moya, I. (2007). Prevalence of mental disorders in children and adolescents from a Spanish slum. Social Science & Medicine, 64(4), 842–849. https://doi.org/10.1016/j.socscimed.2006.10.031
107	No Study Name	Howell, D. C., Huessy, H. R., & Hassuk, B. (1985). Fifteen‐Year Follow‐up of a Behavioral History of Attention Deficit Disorder. Pediatrics, 76(2), 185–190. https://doi.org/10.1542/peds.76.2.185
108	Northern Finland Birth Cohort 1986	Northern Finland Birth Cohorts | University of Oulu. (2022, August 7). https://www.oulu.fi/en/university/faculties‐and‐units/faculty‐medicine/northern‐finland‐birth‐cohorts‐and‐arctic‐biobank/research‐program‐health‐and‐well‐being
109	Northern Swedish Cohort	Hammarström, A., & Janlert, U. (2012). Cohort Profile: The Northern Swedish Cohort. International Journal of Epidemiology, 41(6), 1545–1552. https://doi.org/10.1093/ije/dyr118
110	Norwegian Mother and Child Cohort Study (MoBa)	Magnus, P., Irgens, L. M., Haug, K., Nystad, W., Skjærven, R., Stoltenberg, C., & The Moba Study Group. (2006). Cohort profile: The Norwegian Mother and Child Cohort Study (MoBa). International Journal of Epidemiology, 35(5), 1146–1150. https://doi.org/10.1093/ije/dyl170
111	NU/UCLA Youth Emotion Project	Youth Emotion Project—Anxiety and Depression Research Center at UCLA. (2022, August 7). https://anxietydepression.psych.ucla.edu/youth‐emotion‐project
112	Odense Child Cohort	Kyhl, H. B., Jensen, T. K., Barington, T., Buhl, S., Norberg, L. A., Jørgensen, J. S., Jensen, D. F. G., Christesen, H. T., Lamont, R. F., & Husby, S. (2015). The Odense Child Cohort: Aims, design, and cohort profile. Paediatric and Perinatal Epidemiology, 29(3), 250–258. https://doi.org/10.1111/ppe.12183
113	Ontario Child Health Study	Boyle, M. H., Offord, D. R., Hofmann, H. G., Catlin, G. P., Byles, J. A., Cadman, D. T., Crawford, J. W., Links, P. S., Rae‐Grant, N. I., & Szatmari, P. (1987). Ontario Child Health Study: I. Methodology. Archives of General Psychiatry, 44(9), 826–831. https://doi.org/10.1001/archpsyc.1987.01800210078012
114	Oregon Adolescent Depression Project (OADP)	Lewinsohn, P. M., Hops, H., Roberts, R. E., Seeley, J. R., & Andrews, J. A. (1993). Adolescent psychopathology: I. Prevalence and incidence of depression and other DSM‐III—R disorders in high school students. Journal of Abnormal Psychology, 102(1), 133–144. https://doi.org/10.1037/0021‐843X.102.1.133
115	Panel Study of Income Dynamics' Child Development Supplement and Transition into Adulthood Study	McGonagle, K. A., & Sastry, N. (2015). Cohort Profile: The Panel Study of Income Dynamics' Child Development Supplement and Transition into Adulthood Study. International Journal of Epidemiology, 44(2), 415–422. https://doi.org/10.1093/ije/dyu076
116	Panel Study on Korean Children (PSKC)	Annual Study | KICCE. (2022, August 7). https://panel.kicce.re.kr/engpskc/html.do?menu_idx=13
117	Port Pirie Cohort study	Searle, A. K., Baghurst, P. A., van Hooff, M., Sawyer, M. G., Sim, M. R., Galletly, C., Clark, L. S., & McFarlane, A. C. (2014). Tracing the long‐term legacy of childhood lead exposure: A review of three decades of the Port Pirie Cohort study. NeuroToxicology, 43, 46–56. https://doi.org/10.1016/j.neuro.2014.04.004
118	Prediction and prevention of preeclampsia and intrauterine growth restriction (PREDO) study	Girchenko, P., Lahti, M., Tuovinen, S., Savolainen, K., Lahti, J., Binder, E. B., Reynolds, R. M., Entringer, S., Buss, C., Wadhwa, P. D., Hämäläinen, E., Kajantie, E., Pesonen, A.‐K., Villa, P. M., Laivuori, H., & Räikkönen, K. (2017). Cohort Profile: Prediction and prevention of preeclampsia and intrauterine growth restriction (PREDO) study. International Journal of Epidemiology, 46(5), 1380–1381g. https://doi.org/10.1093/ije/dyw154
119	Project EAT	About—Project EAT ‐ School of Public Health—University of Minnesota. (2022a, August 7). School of Public Health. https://www.sph.umn.edu/research/projects/project‐eat/about/
120	Project on Human Development in Chicago Neighborhoods	PHDCN | Longitudinal Cohort Study | NACJD. (2022, August 7). https://www.icpsr.umich.edu/web/pages/NACJD/guides/phdcn/lcs.html
121	Project to Learn About ADHD in Youth (PLAY)	McKeown, R. E., Holbrook, J. R., Danielson, M. L., Cuffe, S. P., Wolraich, M. L., & Visser, S. N. (2015). The impact of case definition on attention‐deficit/hyperactivity disorder prevalence estimates in community‐based samples of school‐aged children. Journal of the American Academy of Child and Adolescent Psychiatry, 54(1), 53–61. https://doi.org/10.1016/j.jaac.2014.10.014
122	Quebec Longitudinal Study of Child Development (QLSCD)	Orri, M., Boivin, M., Chen, C., Ahun, M. N., Geoffroy, M.‐C., Ouellet‐Morin, I., Tremblay, R. E., & Côté, S. M. (2021). Cohort Profile: Quebec Longitudinal Study of Child Development (QLSCD). Social Psychiatry and Psychiatric Epidemiology, 56(5), 883–894. https://doi.org/10.1007/s00127‐020‐01972‐z
123	Quebec Longitudinal Study of Kindergarten Children (QLSKC)	Rouquette, A., Cote, S. M., Pryor, L. E., Carbonneau, R., Vitaro, F., & Tremblay, R. E. (2014). Cohort Profile: The Quebec Longitudinal Study of Kindergarten Children (QLSKC). International Journal of Epidemiology, 43(1), 23–33. https://doi.org/10.1093/ije/dys177
124	Research with East London Adolescents: Community Health Survey (RELACHS)	Clark, C., Haines, M. M., Head, J., Klineberg, E., Arephin, M., Viner, R., Taylor, S. J. C., Booy, R., Bhui, K., & Stansfeld, S. A. (2007). Psychological symptoms and physical health and health behaviours in adolescents: A prospective 2‐year study in East London. Addiction, 102(1), 126–135. https://doi.org/10.1111/j.1360‐0443.2006.01621.x
125	Resilience, Ethnicity and AdolesCent Mental Health (REACH) study	Knowles, G., Gayer‐Anderson, C., Beards, S., Blakey, R., Davis, S., Lowis, K., Stanyon, D., Ofori, A., Turner, A., Group, S. W., Pinfold, V., Bakolis, I., Reininghaus, U., Harding, S., & Morgan, C. (2021). Mental distress among young people in inner cities: The Resilience, Ethnicity and AdolesCent Mental Health (REACH) study. J Epidemiol Community Health, 75(6), 515–522. https://doi.org/10.1136/jech‐2020‐214315
126	Rhea Mother Child Cohort	Chatzi, L., Leventakou, V., Vafeiadi, M., Koutra, K., Roumeliotaki, T., Chalkiadaki, G., Karachaliou, M., Daraki, V., Kyriklaki, A., Kampouri, M., Fthenou, E., Sarri, K., Vassilaki, M., Fasoulaki, M., Bitsios, P., Koutis, A., Stephanou, E. G., & Kogevinas, M. (2017). Cohort Profile: The Mother‐Child Cohort in Crete, Greece (Rhea Study). International Journal of Epidemiology, 46(5), 1392–1393k. https://doi.org/10.1093/ije/dyx084
127	ROOTS project	Goodyer, I. M., Croudace, T., Dunn, V., Herbert, J., & Jones, P. B. (2010). Cohort Profile: Risk patterns and processes for psychopathology emerging during adolescence: the ROOTS project. International Journal of Epidemiology, 39(2), 361–369. https://doi.org/10.1093/ije/dyp173
128	Shandong Adolescent Behavior and Health Cohort (SABHC)	Liu, B.‐P., Liu, Z.‐Z., Wang, Z.‐Y., An, D., Wei, Y.‐X., Liu, X., & Jia, C. (2019). 0803 Sleep Duration and Depressive Symptoms in Chinese Adolescents: A 3‐wave Prospective Cohort Study. Sleep, 42(Supplement_1), A322–A323. https://doi.org/10.1093/sleep/zsz067.801
129	Taiwan Adolescent to Adult Longitudinal Study (TAALS)	Chien, Y.‐N., Chen, P.‐L., Chen, Y.‐H., Chang, H.‐J., Yang, S.‐C., Chen, Y. C., & Chiou, H.‐Y. (2018). The Taiwan Adolescent to Adult Longitudinal Study (TAALS): Methodology and Cohort Description. Asia Pacific Journal of Public Health, 30(2), 188–197. https://doi.org/10.1177/1010539517754017
130	Taiwan Birth Cohort Study (TBCS)	Chang, L.‐Y., Lin, Y.‐H., Lin, S.‐J., & Chiang, T. (2021). Cohort Profile: Taiwan Birth Cohort Study (TBCS). International Journal of Epidemiology, 50(5), 1430–1431i. https://doi.org/10.1093/ije/dyab048
131	Taiwan Education Panel Survey (TEPS)	About TEPS—台灣教育長期追蹤資料庫後續調查. (2022, August 7). https://tepsb.nccu.edu.tw/about‐teps/
132	Tokyo Teen Cohort (TTC) study/Tokyo TEEN Cohort/Tokyo Early Adolescence Survey (T‐EAS)	Ando, S., Nishida, A., Yamasaki, S., Koike, S., Morimoto, Y., Hoshino, A., Kanata, S., Fujikawa, S., Endo, K., Usami, S., Furukawa, T. A., Hiraiwa‐Hasegawa, M., Kasai, K., & TTC Scientific and Data Collection Team. (2019). Cohort Profile: The Tokyo Teen Cohort study (TTC). International Journal of Epidemiology, 48(5), 1414–1414g. https://doi.org/10.1093/ije/dyz033
133	Trondheim Early Secure Study (TESS)	Steinsbekk, S., & Wichstrøm, L. (2018). Cohort Profile: The Trondheim Early Secure Study (TESS)—a study of mental health, psychosocial development and health behaviour from preschool to adolescence. International Journal of Epidemiology, 47(5), 1401–1401i. https://doi.org/10.1093/ije/dyy190
134	Unbiased Recognition of gaming disorder in Early adolescence (iCURE)	Jeong, H., Yim, H. W., Jo, S.‐J., Lee, S.‐Y., Kim, E., Son, H. J., Han, H., Lee, H. K., Kweon, Y.‐S., Bhang, S., Choi, J.‐S., Kim, B.‐N., Gentile, D. A., & Potenza, M. N. (2017). Study protocol of the internet user Cohort for Unbiased Recognition of gaming disorder in Early adolescence (iCURE), Korea, 2015–2019. BMJ Open, 7(10), e018350. https://doi.org/10.1136/bmjopen‐2017‐018350
135	Victoria Healthy Youth Survey	Leadbeater, B., Stanwick, R., Fyfe, M., & Sukhawathanakul, P. (2016). Changes and Challenges: A Decade of Observations of the Health and Well‐Being of Young Adults in British Columbia. https://onlineacademiccommunity.uvic.ca/vhys/wp‐content/uploads/sites/1967/2016/08/indicators‐report‐aug‐13‐16‐links‐in‐intro‐compressed.pdf
136	Victorian Adolescent Health Cohort Study (VAHCS)	Farrell, T. (2022, August 7). 2000 Stories—Murdoch Children's Research Institute. https://www.mcri.edu.au/research/projects/2000‐stories
137	West Jutland Cohort Study (VestLiv)	Resultater fra VestLiv. (2022, August 7). https://www.arbejdsmedicin.rm.dk/forskning/ulighed‐i‐sundhed/vestliv/resultater‐fra‐VestLiv/
138	Western Australian Pregnancy Cohort/Raine Study	The Raine Study 22 year follow‐up Investigator Group, Straker, L. M., Hall, G. L., Mountain, J., Howie, E. K., White, E., McArdle, N., & Eastwood, P. R. (2015). Rationale, design and methods for the 22 year follow‐up of the Western Australian Pregnancy Cohort (Raine) Study. BMC Public Health, 15(1), 663. https://doi.org/10.1186/s12889‐015‐1944‐6
139	Wirral Child Health and Development Study (WCHADS)	Wirral Child Health and Development Study: First Steps. (2022, August 5). https://www.liverpool.ac.uk/population‐health/research/groups/first‐steps/about/
140	Young in Norway Longitudinal Study	Ung i Norge—Norges første store longitudinelle ungdomsundersøkelse. (2022, August 7). Ung i Norge. https://ung‐i‐norge.no/
141	Young‐HUNT Study	Holmen, T. L., Bratberg, G., Krokstad, S., Langhammer, A., Hveem, K., Midthjell, K., Heggland, J., & Holmen, J. (2014). Cohort profile of the Young‐HUNT Study, Norway: A population‐based study of adolescents. International Journal of Epidemiology, 43(2), 536–544. https://doi.org/10.1093/ije/dys232
142	Youth and Mental Health Study (YAMHS)	Kaasbøll, J., Sigurdson, J. F., Skokauskas, N., & Sund, A. M. (2021). Cohort profile: The Youth and Mental Health Study (YAMHS)—a longitudinal study of the period from adolescence to adulthood. PLOS ONE, 16(2), e0247036. https://doi.org/10.1371/journal.pone.0247036
143	Zurich Epidemiological Studies	Steinhausen, H.‐C., & Winkler Metzke, C. (2003). Prevalence of affective disorders in children and adolescents: Findings from the Zurich Epidemiological Studies. Acta Psychiatrica Scandinavica. Supplementum, 418, 20–23. https://doi.org/10.1034/j.1600‐0447.108.s418.5.x
144	Adolescent Adjustment Project	Adolescent Adjustment Project. (n.d.). Retrieved March 26, 2023, from https://adolescentadjustmentproject.com/about/
145	Asenze Cohort	Chris Desmond, Gabriella A. Norwitz, Jane D. Kvalsvig, Rachel S. Gruver, Shuaib Kauchali, Kathryn G. Watt, Nonhlanhla P. Myeza, Adele Munsami, & Leslie L. Davidson. (2022). The Asenze Cohort Study in KwaZulu‐Natal, South Africa: Protocol and cohort profile. 44, e2022037. https://doi.org/10.4178/epih.e2022037
146	Assessment from Preschool to Puberty‐Longitudinal Epidemiological (APPLE) study	Hirota, T., Adachi, M., Takahashi, M., Mori, H., Shinkawa, H., Sakamoto, Y., Saito, M., & Nakamura, K. (2021). Cohort Profile: The Assessment from Preschool to Puberty—Longitudinal Epidemiological (APPLE) study in Hirosaki, Japan. International Journal of Epidemiology, 50(6), 1782–1783h. https://doi.org/10.1093/ije/dyab112
147	CogBIAS Longitudinal Study	Booth, C., Songco, A., Parsons, S., Heathcote, L. C., & Fox, E. (2019). The CogBIAS longitudinal study of adolescence: Cohort profile and stability and change in measures across three waves. BMC Psychology, 7(1), 73. https://doi.org/10.1186/s40359‐019‐0342‐8
148	FinnBrain Birth Cohort Study	Karlsson, L., Tolvanen, M., Scheinin, N. M., Uusitupa, H.‐M., Korja, R., Ekholm, E., Tuulari, J. J., Pajulo, M., Huotilainen, M., Paunio, T., Karlsson, H., & FinnBrain Birth Cohort Study Group. (2018). Cohort Profile: The FinnBrain Birth Cohort Study (FinnBrain). International Journal of Epidemiology, 47(1), 15–16j. https://doi.org/10.1093/ije/dyx173
149	Future Occupation of Children and Adolescents (FOCA)	Louise Lindholdt, Thomas Lund, Johan Hviid Andersen, Claus D Hansen, & Merete Labriola. (2019). Cohort profile: The Danish Future Occupation of Children and Adolescents cohort (the FOCA cohort): Education, work‐life, health and living conditions in a life‐course perspective. BMJ Open, 9(2), e022784. https://doi.org/10.1136/bmjopen‐2018‐022784
150	Maternal Health Study	Brown, S. J., Gartland, D., Woolhouse, H., Giallo, R., McDonald, E., Seymour, M., Conway, L., FitzPatrick, K. M., Cook, F., Papadopoullos, S., MacArthur, C., Hegarty, K., Herrman, H., Nicholson, J. M., Hiscock, H., & Mensah, F. (2021). The maternal health study: Study design update for a prospective cohort of first‐time mothers and their firstborn children from birth to age 10. Paediatric and Perinatal Epidemiology, 35(5), 612–625. https://doi.org/10.1111/ppe.12757
151	Research and Action for Teens Study (L‐OSDUHS)	Joanna L. Henderson, Leanne K. Wilkins, Lisa D. Hawke, Wei Wang, Marcos Sanches, E.B. Brownlie, & Joseph H. Beitchman. (2021). Longitudinal Emergence of Concurrent Mental Health and Substance Use Concerns in an Ontario School‐Based Sample: The Research and Action for Teens Study. 30(4), 249–263.
152	Stress, development and mental health study (TAM)	Noora Berg, Olli Kiviruusu, Jenna Grundström, Taina Huurre, & Mauri Marttunen. (2021). Stress, development and mental health study, the follow‐up study of Finnish TAM cohort from adolescence to midlife: Cohort profile. BMJ Open, 11(12), e046654. https://doi.org/10.1136/bmjopen‐2020‐046654
153	Surrey Communication and Language in Education Study (SCALES)	SCALES. (2023, March 26). http://www.lilac‐lab.org/scales/scales/
154	Taiwan Youth Project	Wang, Y.‐C. L., Chan, H.‐Y., & Chen, P.‐C. (2018). Transitions of Developmental Trajectories of Depressive Symptoms Between Junior and Senior High School Among Youths in Taiwan: Linkages to Symptoms in Young Adulthood. Journal of Abnormal Child Psychology, 46(8), 1687–1704. https://doi.org/10.1007/s10802‐018‐0408‐8
155	Three Cities Study	Mörtberg, E., Jansson Fröjmark, M., Van Zalk, N., & Tillfors, M. (2022). A longitudinal study of prevalence and predictors of incidence and persistence of sub‐diagnostic social anxiety among Swedish adolescents. Nordic Psychology, 74(3), 152–170. https://doi.org/10.1080/19012276.2021.1943498
156	Toledo Adolescent Relationships Study	Giordano, P. C., Longmore, M. A., & Manning, W. D. (2011). Toledo Adolescent Relationships Study (TARS): Wave 1, 2001 [Data set]. Inter‐university Consortium for Political and Social Research [distributor]. https://doi.org/10.3886/ICPSR04679.v1
157	Trends in Tobacco Use Survey (TITUS)	Trends in Tobacco Use Study (TITUS). (n.d.). Retrieved March 26, 2023, from https://centerforpopulationhealth.usc.edu/?v=Z2E1R1ZhR0RLSlNYY20yRUFXNzgwOU1KNWRXTk52ZnJLeXJkYmxQMXFOTklEUjR6a1hEcHhmR1FOYXVJSlFNYg==
158	Understanding the Lives of Adolescents and Young Adults (UDAYA)	UDAYA. (n.d.). Retrieved March 26, 2023, from https://www.projectudaya.in/
159	Youth Development Survey	Mortimer, J. T. (2021). Youth Development Study, 1988–2011 [St. Paul, Minnesota] [Data set]. Inter‐university Consortium for Political and Social Research [distributor]. https://doi.org/10.3886/ICPSR24881.v4

## APPENDIX 5 Systematic review, title and abstract screening of non‐English studies and list of potentially eligible records about non‐English studies

Non‐English language studies resulting from our systematic review were extracted and duplicates removed, resulting in 886 unique records. One researcher (TB) independently reviewed the English titles and abstracts of non‐English records and excluded ineligible records. Ninety four records representing 64 studies were identified from the eligible records. Duplicates and studies already included in our review using English records were removed. The record abstract with the most study information was selected for charting. In total, 34 potentially eligible non‐English studies were identified. Figure A1 shows the PRISMA flow diagram of study identification. Basic study characteristics, including location of study, start and end year, baseline sample size and age at baseline were extracted and are reported in Table A1.

**TABLE A1:** Study characteristics of potentially eligible non‐english studies.

	Study name	Source identified by database search	Language	Start year	End year	Country	Province/State	City	Baseline sample size	Age at baseline
1	Brazil 1997 Birth Cohort	Simoes, V. M. F., Batista, R. F. L., Alves, Ribeiro, C. C. C., Thomaz, E. B. A. F., Carvalho, C. A., Silva, A. A. M. D. (2020). [health of adolescents in the 1997/1998 birth cohort in sao luis, maranhao state, brazil].. Cadernos de Saude Publica, 36(7), e00164519. https://dx.doi.org/10.1590/0102‐311x00164519	Portuguese	1997	2016	Brazil	Maranhao State	Sao Luis	2515	0
2	Bremen Youth Study (BJS)	Groen, G., & Petermann, F. (2005). Depressive Störungen im Jugendalter: Zeitschrift Für Klinische Psychologie Und Psychotherapie, 34(1), 10–18. https://doi.org/10.1026/1616‐3443.34.1.10	German			Germany	Bremen	N/A	1035	12 to 17
3	Child and Adolescent Behaviors in Long‐term Evolution (CABLE)	Chiang Y.‐C., Wu S.‐C. & Yen L.‐L. (2005). Ever having suicide ideation among the 4th graders in northern Taiwan and its correlates. Taiwan Journal of Public Health, 24(6), 471–482. Retrieved from https://ovidsp.ovid.com/ovidweb.cgi?T=JS&PAGE=reference&D=emed9&NEWS=N&AN=43287235.	Chinese	2001	2005	Taiwan	N/A	N/A	2075	4th grade
4	Chinese Adolescent Mental Health Research Program	Hou J. & Chen Z. (2016). The trajectories of adolescent depressive symptoms: Identifying latent subgroups and risk factors. Acta Psychologica Sinica, 48(8), 957–968.	Chinese			China				12.99
5	Integrated Early Childhood Development (IECD)	Wu, T C, Shi, H F, Du, Y F, Zhang, J X, Zhao, C X, Huang, X N, & Wang, X L. (2019). Effect of books and toys on early childhood development in poor rural areas of China. Chinese. 57(3), 187–193. https://doi.org/10.3760/cma.j.issn.0578‐1310.2019.03.006	Chinese	2013		China	Multiple: Shanxi and Guizhou	N/A	2701	0 to 2
6	Kinder in Deutschland (KiD 0–3)	Ulrich, Susanne M, Lochner, Johanna, Walper, Sabine, Ghezih, Sarah & Lux, Ulrike. (2022). The psychosocial stress of families with a child with a developmental delay and existing prevention offers: Results of a longitudinal population study. Kindheit und Entwicklung: Zeitschrift fur Klinische Kinderpsychologie, 31(3), 164–173. https://doi.org/10.1026/0942‐5403/a000384	German	2015	2017	Germany			779	0 to 3
7	Shanghai‐Minhang Birth Cohort Study (S‐MBCS)/China‐Anhui Birth Cohort Study (C‐ABCS)	Fen L., Youping T., Xiaomin L., Ruilan X., Longmei J., Xiaowei S., et al. (2018). A prospective cohort study on the relationship between maternal prenatal depressive symptoms and children's behavioral problems at 2 years old. Chinese Journal of Endemiology, 39(4), 455–459. https://doi.org/10.3760/cma.j.issn.0254‐6450.2018.04.013	Chinese	2012	2014	China	Shanghai	N/A	491	0
8	No Name Identified	Billard, C., Bricout, L., Ducot, B., Richard, G., Ziegler, J., Fluss, J. (2010). [evolution of competence in reading, spelling and comprehension levels in low socioeconomic environments and impact of cognitive and behavioral factors on outcome in two years].. Revue d Epidemiologie et de Sante Publique, 58(2), 101‐10. https://dx.doi.org/10.1016/j.respe.2009.11.002	French	2006		France	Île‐de‐France	Paris	1062	7 to 8
9	No Name Identified	Blanz B., Geisel B. & Laucht M. (1986). The role of the father in the development of school‐age children: Results of an epidemiological study. Zeitschrift fur Kinder‐ und Jugendpsychiatrie, 14(1), 5–31. Retrieved from https://ovidsp.ovid.com/ovidweb.cgi?T=JS&PAGE=reference&D=emed3&NEWS=N&AN=16164883.	German						365	8
10	No Name Identified	Bomba, Jacek & Jaklewicz, Hanna. (1997). Depression in children: A prospective study. Psychiatria Polska, 31(6), 677–689. Retrieved from https://ovidsp.ovid.com/ovidweb.cgi?T=JS&PAGE=reference&D=psyc3&NEWS=N&AN=1998‐00610‐002.	Polish							
11	No Name Identified	Canals Sans, J., Domenech Llaberia, E., Cliville Pages, R., Fernandez Ballart, J., Marti Henneberg, C. (1991). [symptoms of depression during puberty: initial results of a longitudinal epidemiological study].. Actas Luso‐Espanolas de Neurologia, Psiquiatria y Ciencias Afines, , 19(3), 155‐61. Retrieved from https://ovidsp.ovid.com/ovidweb.cgi?T=JS&PAGE=reference&D=med3&NEWS=N&AN=1950698.	Spanish						534	11 to 12
12	No Name Identified	Canals, J, Fernandez‐Ballart, J & Marti‐Henneberg, C. (1992). Epidemiology of depressive symptomatology in a sample of adolescents: A four years follow‐up. Revista de Psiquiatria de la Facultad de Medicina de Barcelona, 19(2), 73–81. Retrieved from https://ovidsp.ovid.com/ovidweb.cgi?T=JS&PAGE=reference&D=psyc3&NEWS=N&AN=1992‐87756‐001.	Spanish			Spain	N/A	N/A	336	11 to 15
13	No Name Identified	Chiland, C., Coppel, L., Coumes, F., Diatkine, R., Gabel, M. (1966). [epidemiologic information furnished by the longitudinal study of a group of children from the schools of the 13th district of paris].. Bulletin de l'Institut National de la Sante et de la Recherche Medicale, 21(3), 455‐66. Retrieved from https://ovidsp.ovid.com/ovidweb.cgi?T=JS&PAGE=reference&D=med1&NEWS=N&AN=5941716.	French			France	Île‐de‐France	Paris		
14	No Name Identified	Wang, Kai & Su, Lin‐yan. (2005). A Two Year Follow‐up Study of Anxiety Disorders in Children Aged 7–10. 13(2), 173–176.	Chinese			China	Hunan	Changsha	206	8.72+/−0.92
15	No Name Identified	Morgenstern, M., Isensee, B., & Hanewinkel, R. (2019). Vorhersage des Rauschtrinkens im jungen Erwachsenenalter: Eine Kohortenstudie über 9 Jahre [Prediction of binge drinking in young adults: a cohort study over nine years]. Zeitschrift fur Kinder‐ und Jugendpsychiatrie und Psychotherapie, 47(2), 112–124. https://doi.org/10.1024/1422‐4917/a000590	German	2006	2015	Germany	Schleswig‐Holstein and Saxony‐Anhalt	N/A	5176	12.6
16	No Name Identified	Madeddu, F., Dazzi, S., Prunas, A., Ripamonti, C., & Barzaghi, A. (2006). Prevalence of Attention Deficit/Hyperactivity Disorder (DSM‐III‐R) in a large sample of Italian preadolescents. Minerva Psichiatrica, 47, 209–219.	Italian			Italy	Parma	N/A	570	11 to 12
17	No Name Identified	Gordillo, R., Del Barrio, V., & Carrasco, M. Á. (2012). Análisis longitudinal de la comorbilidad entre depresión y agresión: Cronicidad y severidad en sujetos de 11 a 13 años [Longitudinal analysis of comorbidity between depression and aggression chronicity and severity in individuals 11–13 years]. Interdisciplinaria Revista de Psicología y Ciencias Afines, 29(1), 165–185. https://doi.org/10.16888/interd.2012.29.1.10	Spanish			Spain	Madrid	Madrid	525	11 to 13
18	No Name Identified	De La Barra F., Toledo V. & Rodriguez J. (2003). Mental health study in two cohorts of schoolchildren from west Santiago. III: Early predictors of behavioral and cognitive problems. Revista Chilena de Neuro‐Psiquiatria, 41(1), 65–74. Retrieved from https://ovidsp.ovid.com/ovidweb.cgi?T=JS&PAGE=reference&D=emed8&NEWS=N&AN=43161475.	Spanish	1992	1997	Chile	Santiago	Multiple: Pudahuel, Cerro Navia and Lo Prado	1279	Grade 1
19	No Name Identified	Fujita, T., Kurisu, E., Haratani, T., Kim, Y., Asakura, T., Asakura, Y. (1990). [a six‐year follow‐up study of childhood behavior problems].. Nippon Koshu Eisei Zasshi ‐ Japanese Journal of Public Health, 37(2), 57–66. Retrieved from https://ovidsp.ovid.com/ovidweb.cgi?T=JS&PAGE=reference&D=med3&NEWS=N&AN=2131969.	Japanese	1977	1983	Japan	Tokyo	Bunkyo	279	3 to 7
20	No Name Identified	Garcia‐Jimenez, M. C., Lopez‐Pison, J., Blasco‐Arellano, M. M. (2005). [the primary care paediatrician in attention deficit hyperactivity disorder. an approach involving a population study].. Revista de Neurologia, 41(2), 75–80. Retrieved from https://ovidsp.ovid.com/ovidweb.cgi?T=JS&PAGE=reference&D=med6&NEWS=N&AN=16028184.	Spanish			Spain	Navarre	Multiple: Bunuel and Cortes		6 tp 12
21	No Name Identified	Gisselmann, A., Pinoit, J. M. (1995). [manic‐depressives, time of birth, time of onset. an epidemiological study].. Annales Medico‐Psychologiques, 153(10), 707‐11. Retrieved from https://ovidsp.ovid.com/ovidweb.cgi?T=JS&PAGE=reference&D=med3&NEWS=N&AN=8720365.	French							
22	No Name Identified	Gong, C., Fang, J., Shan, J., Duan, X. N., Hu, J. J., Chen, H. R., Zhang, J. J., Wan, Y. H., Sun, Y. (2018). [prospective association between childhood abuse experiences and depressive symptoms in adolescence].. Chung‐Hua Liu Hsing Ping Hsueh Tsa Chih Chinese Journal of Epidemiology, 39(9), 1184–1187. https://dx.doi.org/10.3760/cma.j.issn.0254‐6450.2018.09.008	Chinese	2013	2017	China	Anhui province	Bengbu	1172	Grade 3 and 4
23	No Name Identified	Lebedev, M. A. (1997). [premorbid states in borderline mental disorders].. Zhurnal Nevrologii i Psikhiatrii Imeni S.S. Korsakova, 97(6), 22‐5. Retrieved from https://ovidsp.ovid.com/ovidweb.cgi?T=JS&PAGE=reference&D=med4&NEWS=N&AN=11517471.	Russian							14 to 26
24	No Name Identified	Modrzejewska R., Bomba J., Cofor P. & Pac A. (2022). Depressive symptoms in adolescence and quality of life 17 years later ‐ follow‐up study. Psychiatria Polska, 56(1), 51–61. https://doi.org/10.12740/PP/OnlineFirst/124273	Polish						3445	
25	No Name Identified	Pires T.O., da Silva C.M.F.P. & de Assis S.G. (2012). Family environment and attentiondeficit hyperactivity disorder. Revista de Saude Publica, 46(4), 624–633. https://doi.org/10.1590/S0034‐89102012005000043	Portuguese	2005	2008	Brazil	Rio de Janeiro	Sao Goncalo	479	Grade 2
26	No Name Identified	Richard, R., Marcotte, D. (2013). [the temporal relationship between anxiety and depression during the school transition between primary and high‐school].. Sante Mentale au Quebec, 38(2), 257‐75.	French	2003	2012	Canada	Quebec		499	11.22
27	No Name Identified	Schulz, W., Vormberg, J., Hahlweg, K. (2021). [how do adolescents see their parents? prevalences, predictors and relationships in longitudinal and cross‐section research].. Praxis der Kinderpsychologie und Kinderpsychiatrie, 70(3), 198–216. https://dx.doi.org/10.13109/prkk.2021.70.3.198	German						343	14
28	No Name Identified	Su, C., Lai, S., Song, S., Xu, H., Liu, Q. (2020). [influencing factors of depressive symptoms of rural middle school students in zizhong county based on two‐level logistic regression model].. Wei Sheng Yen Chiu/Journal of Hygiene Research, 49(3), 427–433. https://dx.doi.org/10.19813/j.%20cnki.%20weishengyanjiu. 2020. 03. 014	Chinese	2015		China	Sichuan Province	Zizhong County	4934	Middle school and high school students
29	No Name Identified	Suzuki K., Matsushita S., Kimura M., Takeda A. & Higuchi S. (2011). [Results of 10‐year cohort study on Japanese adolescent drinking]. Nihon Arukoru Yakubutsu Igakkai zasshi = Japanese journal of alcohol studies & drug dependence, 46(5), 470–485. Retrieved from https://ovidsp.ovid.com/ovidweb.cgi?T=JS&PAGE=reference&D=emed12&NEWS=N&AN=364229790.	Japanese	1997	2007	Japan	Kanagawa prefecture	N/A	802	13.5
30	No Name Identified	Suzuki, K., Takeda, A., Matsushita, S., Higuchi, S., Shirakura, K. (2002). [a cohort study of japanese adolescent alcohol use and misuse (1): observation for 2 years].. Nihon Arukoru Yakubutsu Igakkai Zasshi, 37(6), 577‐85. Retrieved from https://ovidsp.ovid.com/ovidweb.cgi?T=JS&PAGE=reference&D=med4&NEWS=N&AN=12607945.	Japanese	1997		Japan	N/A	N/A	629	Grade 7 to 9 (M: 13.5)
31	No Name Identified	Waligora, Katja. (2003). Norm‐breaking behavior, physical disorders, and depressive symptoms of students‐What role does social support play? Kindheit und Entwicklung: Zeitschrift fur Klinische Kinderpsychologie, 12(3), 145–153. https://doi.org/10.1026//0942‐5403.12.3.145	German						326	10 to 15
32	No Name Identified	Wang X., Sun Y., An J., Hao J. & Tao F. (2013). Gender difference on depressive symptoms among Chinese children and adolescents. Zhonghua liu xing bing xue za zhi = Zhonghua liuxingbingxue zazhi, 34(9), 893–896. Retrieved from https://ovidsp.ovid.com/ovidweb.cgi?T=JS&PAGE=reference&D=emed14&NEWS=N&AN=603934131.	Chinese			China	N/A	N/A		9 to 18
33	No Name Identified	Yuge, M., Zen, Y. (2009). [a follow‐up study of 6‐year‐old kindergarten children at one year after they participated in a pilot medical examination].. No to Hattatsu [Brain & Development], 41(4), 269‐74. Retrieved from https://ovidsp.ovid.com/ovidweb.cgi?T=JS&PAGE=reference&D=med7&NEWS=N&AN=19618882.	Japanese	2007	2008				129	6
34	No Name Identified	Zhao, X., Yang, L., Chen, M., Chen, J., Lyu, X., Jiang, Y., Sun, Y., Sun, Y. (2014). [depressive symptoms and related factors among primary and middle school students in changfeng county of anhui province:a two‐year longitudinal study].. Chung‐Hua Liu Hsing Ping Hsueh Tsa Chih Chinese Journal of Epidemiology, 35(5), 505‐9. Retrieved from https://ovidsp.ovid.com/ovidweb.cgi?T=JS&PAGE=reference&D=med11&NEWS=N&AN=25059356.	Chinese	2009	2011	China	Anhui province	Changfeng county	816	Grade 3 to 9

## APPENDIX 6 List of records that could not be excluded based on our exclusion criteria but had no information source.

**Table:**

	Study name	Record from database search
1	BROMS	Wirback, T., Möller, J., Larsson, J.‐O., Galanti, M. R., & Engström, K. (2014). Social factors in childhood and risk of depressive symptoms among adolescents—A longitudinal study in Stockholm, Sweden. International Journal for Equity in Health, 13(1), 96. https://doi.org/10.1186/s12939‐014‐0096‐0
2	Chinese Adolescents Health Survey	Guo L., Wang W., Wang T., Zhao M., Wu R. & Lu C. (2021). The Longitudinal Association between Sleep Duration and Suicidal Behavior among Chinese Adolescents: The Role of Nonmedical Use of Prescription Drug. Behavioral sleep medicine, 19(5), 589–601. https://doi.org/10.1080/15402002.2020.1822361
3	Chinese Longitudinal	Chen C. (2022). Trajectories of Behavioral Problems and Predictors in Children Aged 4–7 Years: a Growth Mixture Model Analysis. Journal of Psychopathology and Behavioral Assessment, 44(3), 636–648. https://doi.org/10.1007/s10862‐022‐09953‐z
	Study of Rearing and Child Development (CLSRCD)	
4	Cleveland TeenZzz/Cleveland Children's Sleep and Health Study (CCSHS)	Moore, M., Kirchner, H. L., Drotar, D., Johnson, N., Rosen, C., Ancoli‐Israel, S., & Redline, S. (2009). Relationships Among Sleepiness, Sleep Time, and Psychological Functioning in Adolescents. Journal of Pediatric Psychology, 34(10), 1175–1183. https://doi.org/10.1093/jpepsy/jsp039
5	Dating It Safe	Mori C., Choi H.J., Temple J.R. & Madigan S. (2021). Patterns of sexting and sexual behaviors in youth: A Latent Class Analysis. Journal of Adolescence, 88 97–106. https://doi.org/10.1016/j.adolescence.2021.01.010
6	Facing Rejection Project	Wang Y., Hawk S.T., Branje S. & Van Lissa C.J. (2022). Longitudinal links between expressive flexibility and friendship quality in adolescence: The moderating effect of social anxiety. Journal of adolescence, no pagination. https://doi.org/10.1002/jad.12123
7	Flourishing Families Project (FFP)	Rollins, Elizabeth Mathews & Crandall, AliceAnn. (2021). Self‐regulation and shame as mediators between childhood experiences and young adult health. Frontiers in Psychiatry, 12 https://doi.org/10.3389/fpsyt.2021.649911
8	Gene Environment Mood (GEM) study	Hankin, B. L., Young, J. F., Abela, J. R. Z., Smolen, A., Jenness, J. L., Gulley, L. D., Technow, J. R., Gottlieb, A. B., Cohen, J. R., & Oppenheimer, C. W. (2015). Depression from childhood into late adolescence: Influence of gender, development, genetic susceptibility, and peer stress. Journal of Abnormal Psychology, 124(4), 803–816. https://doi.org/10.1037/abn0000089
9	GenerationFRee survey	Barrense‐Dias, Y., Akre, C., Berchtold, A., & Suris, J.‐C. (2018). Gambling or Not Gambling: What Makes the Difference? Journal of Adolescent Health, 62, S125. https://doi.org/10.1016/j.jadohealth.2017.11.255
10	Healthy Teens Longitudinal Study	Orpinas, P., Nahapetyan, L., & Truszczynski, N. (2017). Low and Increasing Trajectories of Perpetration of Physical Dating Violence: 7‐Year Associations with Suicidal Ideation, Weapons, and Substance Use. Journal of Youth and Adolescence, 46(5), 970–981. https://doi.org/10.1007/s10964‐017‐0630‐7
11	Kauai Longitudinal Study	Werner, E. E., & Smith, R. S. (1979). An epidemiologic perspective on some antecedents and consequences of childhood mental health problems and learning disabilities: a report from the Kauai Longitudinal Study. Journal of the American Academy of Child Psychiatry, 18(2), 292–306. https://ovidsp.ovid.com/ovidweb.cgi?T=JS&PAGE=reference&D=med1&NEWS=N&AN=447961
12	Koshu Project/Project Enzan	Sato, M., Nagai, A., Suzuki, K., Tanaka, T., Kondo, N., & Yamagata, Z. (2010). Association between the trajectories of weight status and depression in puberty. American Journal of Epidemiology, 171(SUPPL. 11), S11. https://dx.doi.org/10.1093/aje/kwq151 (43rd Annual Meeting of the Society for Epidemiologic Research, SER 2010. Anaheim, CA United States.
13	Kyushu Okinawa Maternal and Child Health Study	Miyake, Y., Tanaka, K., Okubo, H., Sasaki, S., & Arakawa, M. (2020). Maternal B vitamin intake during pregnancy and childhood behavioral problems in Japan: The Kyushu Okinawa Maternal and Child Health Study. Nutritional Neuroscience, 23(9), 706–713. https://dx.doi.org/10.1080/1028415X.2018.1548139
14	Longhua Child Cohort Study	Fang, X.‐Y., Liu, L., Jiang, H., Strodl, E., Liu, B.‐Q., Yin, X.‐N., Wen, G.‐M., Sun, D.‐L., Xian, D.‐X., Wu, C.‐A., Jing, J., Jin, Y., & Chen, W.‐Q. (2019). Association between prenatal exposure to household inhalants exposure and ADHD‐like behaviors at around 3 years of age: Findings from Shenzhen Longhua Child Cohort Study. Environmental Research, 177, 108612. https://dx.doi.org/10.1016/j.envres.2019.108612
15	Longitudinal Study of Adolescents' Mental and Behavioral Well‐being Research (LSAMBR)	Wang W., Du X., Guo Y., Li W., Teopiz K.M., Shi J., et al. (2021). The associations between sleep situations and mental health among Chinese adolescents: A longitudinal study. Sleep Medicine, 82 71–77. https://doi.org/10.1016/j.sleep.2021.03.009
16	Longitudinal Study of Children's and Adolescents' Family and Social Experiences (LSCAFSE)	Hsieh Y.‐P., Hwa H.‐L., Shen A.C.‐T., Wei H.‐S., Feng J.‐Y. & Huang C.‐Y. (2021). Ecological predictors and trajectory of internet addiction from childhood through adolescence: a nationally representative longitudinal study. International Journal of Environmental Research and Public Health, 18(12), no pagination. https://doi.org/10.3390/ijerph18126253
17	Ma'anshan Birth Cohort study (MABC)	Zhu B., Deng F., Yan S., Huang K., Wu X., Tao X., et al. (2021). Gestational diabetes mellitus, autistic traits and ADHD symptoms in toddlers: Placental inflammatory and oxidative stress cytokines do not play an intermediary role. Psychoneuroendocrinology, 134 no pagination. https://doi.org/10.1016/j.psyneuen.2021.105435
18	Maternal Health Practices and Child Development Project	Baraban, E. A. (2003). The effect of maternal depressive symptoms on adolescent smoking behavior [Health Psychology & Medicine 3360]. Dissertation Abstracts International: Section B: The Sciences and Engineering, 64(1‐B), 140. https://ovidsp.ovid.com/ovidweb.cgi?T=JS&PAGE=reference&D=psyc4&NEWS=N&AN=2003‐95014‐295 (Dissertation Abstracts International)
19	MATQUID (Quid of Maternity	Sutter‐Dallay, A. (2015). The matquid cohort: Links between maternal depressive symptoms and children's developmental trajectories. European Psychiatry, 30(SUPPL. 1), 106. https://ovidsp.ovid.com/ovidweb.cgi?T=JS&PAGE=reference&D=emed16&NEWS=N&AN=71930945 (23rd European Congress of Psychiatry, EPA 2015. Vienna Austria.
20	National Survey of Children's Exposure to Violence	Turner, H. A., Finkelhor, D., Shattuck, A., & Hamby, S. (2012). Recent victimization exposure and suicidal ideation in adolescents. Archives of Pediatrics and Adolescent Medicine, 166(12), 1149–1154. https://dx.doi.org/10.1001/archpediatrics.2012.1549
21	Norwegian Arctic Adolescent Health Study	Eckhoff, C., Straume, B., & Kvernmo, S. (2017). Multisite musculoskeletal pain in adolescence and later mental health disorders: a population‐based registry study of Norwegian youth: the NAAHS cohort study. BMJ Open, 7(2), e012035. https://dx.doi.org/10.1136/bmjopen‐2016‐012035
22	PELAGIE birth cohort	Costet, N., Beranger, R., Garlantezec, R., Rouget, F., Monfort, C., Cordier, S., Pele, F., & Chevrier, C. (2018). Occupational exposure to organic solvents during pregnancy and childhood behavior: findings from the PELAGIE birth cohort (France, 2002–2013). Environmental health: a global access science source, 17(1), 63. https://dx.doi.org/10.1186/s12940‐018‐0406‐x (Erratum in: Environ Health. 2018 Sep 4; 17(1):71; PMID: 30180859 [https://www.ncbi.nlm.nih.gov/pubmed/30180859])
23	Piedmont Health Survey	Turnbull, J. E., George, L. K., Landerman, R., Swartz, M. S., & Blazer, D. G. (1990). Social outcomes related to age of onset among psychiatric disorders. Journal of Consulting and Clinical Psychology, 58(6), 832–839. https://ovidsp.ovid.com/ovidweb.cgi?T=JS&PAGE=reference&D=med3&NEWS=N&AN=2292633
24	PREDICT Resilience longitudinal cohort	Songtaweesin, W. N., Puthanakit, T., Chandra, C., Sophonphan, J., Sun, L. P., Ounchanum, P., Kosalaraksa, P., Aurpibul, L., Kanjanavanit, S., Ngampiyaskul, C., Malee, K., Paul, R., Ananworanich, J., & Mellins, C. A. (2020). Depression associated with lower executive functioning in Asian youth living with HIV. Topics in Antiviral Medicine, 28(1), 310–311. https://ascopubs.org/doi/abs/10.1200/JCO.2020.38.29_suppl.832
25	Rutgers Health and Human Development Project	Warner, L. A., White, H. R., & Johnson, V. (2007). Alcohol initiation experiences and family history of alcoholism as predictors of problem‐drinking trajectories. Journal of Studies on Alcohol and Drugs, 68(1), 56–65. https://ovidsp.ovid.com/ovidweb.cgi?T=JS&PAGE=reference&D=med6&NEWS=N&AN=17149518
26	Shanghai Maternal–Child Pairs Cohort (Shanghai MCPC)	Zhang, S., Ma, X., Wei, Q., Zhang, Y., Wang, L., Shi, H. (2022). Maternal pre‐pregnancy bmi and gestational weight gain modified the association between prenatal depressive symptoms and toddler's emotional and behavioral problems: a prospective cohort study.. Nutrients, 15(1). https://dx.doi.org/10.3390/nu15010181
27	South London Child Development Study	Bauer, A., Pawlby, S., Plant, D. T., King, D., Pariante, C. M., & Knapp, M. (2015). Perinatal depression and child development: exploring the economic consequences from a South London cohort. Psychological Medicine, 45(1), 51–61. https://dx.doi.org/10.1017/S0033291714001044
28	Stanford Infant Growth Study	Van Tine, M. L., McNicholas, F., Safer, D. L., & Agras, W. S. (2017). Follow‐up of selective eaters from childhood to adulthood. Eating Behaviors, 26, 61–65. https://dx.doi.org/10.1016/j.eatbeh.2017.01.003
29	Survey of Adolescent Life in Västmanland Cohort Study (SALVe‐Cohort)	Rebecka K., Cecilia A., Nilsson K.W. & Susanne O. (2021). Gene‐environment interaction: Oxytocin receptor (OXTR) polymorphisms and parenting style as potential predictors for depressive symptoms. Psychiatry Research, 303 no pagination. https://doi.org/10.1016/j.psychres.2021.114057
30	Swedish Seven Schools Longitudinal Study	Ozdemir, M., & Stattin, H. (2011). Bullies, victims, and bully‐victims: A longitudinal examination of the effects of bullying‐victimization experiences on youth well‐being [Behavior Disorders & Antisocial Behavior 3230]. Journal of Aggression, Conflict and Peace Research, 3(2), 97–102. https://dx.doi.org/10.1108/17596591111132918
31	Taiwan Children Health Study	Lin, Y.‐T., Chen, Y.‐C., Gau, S. S.‐F., Yeh, T.‐H., Fan, H.‐Y., Hwang, Y.‐Y., & Lee, Y. L. (2016). Associations between allergic diseases and attention deficit hyperactivity/oppositional defiant disorders in children. Pediatric Research, 80(4), 480–485. https://dx.doi.org/10.1038/pr.2016.111
32	Taiwanese Adolescent Self‐Harm Project	Huang, Y.‐H., Liu, H.‐C., Sun, F.‐J., Liu, S.‐I., Tsai, F.‐J., Huang, K.‐Y., Chen, T.‐C., & Huang, Y.‐P. (2017). Relationship Between Predictors of Incident Deliberate Self‐Harm and Suicide Attempts Among Adolescents. Journal of Adolescent Health, 60(5), 612–618. https://dx.doi.org/10.1016/j.jadohealth.2016.12.005
33	Temporary Assistance for Needy Families in Illinois	Yang, M.‐Y. (2015). The effect of material hardship on child protective service involvement. Child Abuse and Neglect, 41, 113–125. https://dx.doi.org/10.1016/j.chiabu.2014.05.009
34	The Chitwan Valley Family Study	Benjet, C., Axinn, W. G., Hermosilla, S., Schulz, P., Cole, F., Sampson, L., & Ghimire, D. (2020). Exposure to Armed Conflict in Childhood versus Older Ages and Subsequent Onset of Major Depressive Disorder. JAMA Network Open, 3(11), 19848. https://dx.doi.org/10.1001/jamanetworkopen.2020.19848
35	Ulm Birth Cohort Study	Christiansen, H., Brandt, S., Walter, V., Wabitsch, M., Rothenbacher, D., Brenner, H., Schimmelmann, B. G., & Hirsch, O. (2017). Prediction of BMI at age 11 in a longitudinal sample of the Ulm Birth Cohort Study. PLoS ONE, 12(8), e0182338. https://dx.doi.org/10.1371/journal.pone.0182338
36	No Name Identifier	Yuan G. & Liu Z. (2021). Longitudinal cross‐lagged analyses between cyberbullying perpetration, mindfulness and depression among Chinese high school students. Journal of health psychology, 26(11), 1872–1881. https://doi.org/10.1177/1359105319890395
37	No Name Identifier	Wartberg L., Fischer‐Waldschmidt G., Kriston L., Hoven C.W., Sarchiapone M., Carli V., et al. (2021). Longitudinal predictors of problematic alcohol use in adolescence: A 2‐year follow‐up study. Addictive Behaviors, 120 no pagination. https://doi.org/10.1016/j.addbeh.2021.106952
38	No Name Identifier	Zimmer‐Gembeck M.J., Gardner A.A., Hawes T., Masters M.R., Waters A.M. & Farrell L.J. (2021). Rejection sensitivity and the development of social anxiety symptoms during adolescence: A five‐year longitudinal study. International Journal of Behavioral Development, 45(3), 204–215. https://doi.org/10.1177/0165025421995921
39	No Name Identifier	Rapee, R. M., Magson, N. R., Forbes, M. K., Richardson, C. E., Johnco, C. J., Oar, E. L., Fardouly, J. (2022). Risk for social anxiety in early adolescence: longitudinal impact of pubertal development, appearance comparisons, and peer connections.. Behaviour Research & Therapy, 154, 104126. https://dx.doi.org/10.1016/j.brat.2022.104126
40	No Name Identifier	Martinez‐Levy G.A., Campos A.I., Rabinowitz J.A., Garcia‐Marin L.M., Benjet C., Mendez E., et al. (2021). Suicidal ideation and planning among Mexican adolescents are associated with depression polygenic risk scores. American Journal of Medical Genetics, Part B: Neuropsychiatric Genetics, 186(8), 476–484. https://doi.org/10.1002/ajmg.b.32864
41	No Name Identifier	Kojima R., Shinohara R., Akiyama Y., Yokomichi H. & Yamagata Z. (2021). Temporal directional relationship between problematic internet use and depressive symptoms among Japanese adolescents: A random intercept, cross‐lagged panel model. Addictive Behaviors, 120 no pagination. https://doi.org/10.1016/j.addbeh.2021.106989
42	No Name Identifier	Wang, Junyi, Zhou, Yinglu, Ding, Jinhong & Xiao, Jing. (2021). The “weakest link” as an indicator of cognitive vulnerability predicts stress generation: A multi‐wave longitudinal study among adolescents. Journal of Rational‐Emotive & Cognitive‐Behavior Therapy, 39(3), 414–427. https://doi.org/10.1007/s10942‐020‐00380‐1
43	No Name Identifier	Barth Vedoy I., Skulberg K.R., Anderssen S.A., Fagerland M.W., Tjomsland H.E. & Thurston M. (2021). The longitudinal association between objectively measured physical activity and mental health among Norwegian adolescents. International Journal of Behavioral Nutrition and Physical Activity, 18(1), no pagination. https://doi.org/10.1186/s12966‐021‐01211‐x
44	No Name Identifier	Yi X. & Li G. (2021). The longitudinal relationship between internet addiction and depressive symptoms in adolescents: A random‐intercept cross‐lagged panel model. International Journal of Environmental Research and Public Health, 18(24), no pagination. https://doi.org/10.3390/ijerph182412869
45	No Name Identifier	Wuthrich, V. M., Belcher, J., Kilby, C., Jagiello, T., Lowe, C. (2021). Tracking stress, depression, and anxiety across the final year of secondary school: a longitudinal study.. Journal of School Psychology, 88, 18–30. https://dx.doi.org/10.1016/j.jsp.2021.07.004
46	No Name Identifier	Takahashi T., Takebayashi Y., Matsubara K., Inaba Y., Kawasaki Y. & Sato S. (2022). Two‐Year Longitudinal Study of Children's Cognitive Behavioral Characteristics Associated with Depressive Symptoms. Cognitive Therapy and Research, 46(6), 1049–1061. https://doi.org/10.1007/s10608‐022‐10326‐9
47	No Name Identifier	Zhou H., Dang L., Lam L.W., Zhang M.X. & Wu A.M.S. (2021). A cross‐lagged panel model for testing the bidirectional relationship between depression and smartphone addiction and the influences of maladaptive metacognition on them in Chinese adolescents. Addictive Behaviors, 120 no pagination. https://doi.org/10.1016/j.addbeh.2021.106978
48	No Name Identifier	Zhang S., Huang Y., Zaid M. & Tong L. (2022). ADHD Symptoms and Obesity in Chinese Children and Adolescents: A Longitudinal Study With Abnormal Eating Behaviors as Moderating Factors. Journal of attention disorders, 26(11), 1452–1463. https://doi.org/10.1177/10870547221081005
49	No Name Identifier	Ni, N., Chi, X., Liu, W., Cui, X. (2021). Air pollution and adolescent development: evidence from a 3‐year longitudinal study in china.. Children, 8(11). https://dx.doi.org/10.3390/children8110987
50	No Name Identifier	Lai S., Su C., Song S., Yan M., Tang C., Zhang Q., et al. (2021). Depression and Deliberate Self‐Harm Among Rural Adolescents of Sichuan Province in Western China: A 2‐Year Longitudinal Study. Frontiers in Psychiatry, 12 no pagination. https://doi.org/10.3389/fpsyt.2021.605785
51	No Name Identifier	Silva E.P., Emond A. & Ludermir A.B. (2021). Depression in childhood: The role of children's exposure to intimate partner violence and maternal mental disorders. Child Abuse and Neglect, 122 no pagination. https://doi.org/10.1016/j.chiabu.2021.105305
52	No Name Identifier	Yilmaz M., Psychogiou L., Ford T. & Dunn B.D. (2021). Examining the relationship between anhedonia symptoms and trait positive appraisal style in adolescents: A longitudinal survey study. Journal of Adolescence, 91 71–81. https://doi.org/10.1016/j.adolescence.2021.07.007
53	No Name Identifier	Li X., Tang J., Li J., Zhou Y., Wang Y. & Jiang S. (2022). Exposure to domestic violence and depressive symptoms in Chinese adolescents: Sleep problems as a mediator. Journal of Affective Disorders, 310 17–24. https://doi.org/10.1016/j.jad.2022.04.114
